# From kitchen to clinic: Pharmacotherapeutic potential of common spices in Indian cooking in age-related neurological disorders

**DOI:** 10.3389/fphar.2022.960037

**Published:** 2022-11-10

**Authors:** Narges Norouzkhani, Arian Ghannadi Karimi, Negar Badami, Erfan Jalalifar, Behnaz Mahmoudvand, Arina Ansari, Neda Pakrou Sariyarighan, Dorsa Alijanzadeh, Sara Aghakhani, Reza Shayestehmehr, Mohammadreza Arzaghi, Zahra Sheikh, Yasaman Salami, Mohammad Hesam Marabi, Amir Abdi, Niloofar Deravi

**Affiliations:** ^1^ Department of Medical Informatics, Faculty of Medicine, Mashhad University of Medical Sciences, Mashhad, Iran; ^2^ Preclinical, Cardiovascular Imaging Core Facility, Tehran University of Medical Sciences, Tehran, Iran; ^3^ Pharmaceutical Sciences Research Center, Tehran Medical Sciences, Islamic Azad University, Tehran, Iran; ^4^ Student Research Committee, Tabriz University of Medical Sciences, Tabriz, Iran; ^5^ Student Research Committee, School of Medicine, Iran University of Medical Sciences, Tehran, Iran; ^6^ Student Research Committee, School of Medicine, North Khorasan University of Medical Sciences, Bojnurd, Iran; ^7^ School of Medicine, Urmia University of Medical Sciences, Urmia, Iran; ^8^ Student Research committee, School of Medicine, Shahid Beheshti University of Medical Sciences, Tehran, Iran; ^9^ Student Research Committee, Esfahan University of Medical Sciences, Esfahan, Iran; ^10^ Faculty of Veterinary Medicine, Amol University of Special Modern Technologies, Amol, Iran; ^11^ Shahid Beheshti University of Medical Sciences, Tehran, Iran; ^12^ Babol University of Medical Sciences, Babol, Iran; ^13^ Student Research Committee, Kermanshah University of Medical Sciences, Kermanshah, Iran; ^14^ Student Research Committee, School of Medicine, Tehran Medical Sciences, Islamic Azad University, Tehran, Iran

**Keywords:** Indian spices, neurological disorder, aging, complementary medicine, herbal extracts

## Abstract

Aging is described as an advanced time-related collection of changes that may negatively affect with the risk of several diseases or death. Aging is a main factor of several age-related neurological disorders, including neurodegenerative diseases (Alzheimer’s disease, Parkinson’s disease, and dementia), stroke, neuroinflammation, neurotoxicity, brain tumors, oxidative stress, and reactive oxygen species (ROS). Currently available medications for age-related neurological disorders may lead to several side effects, such as headache, diarrhea, nausea, gastrointestinal (GI) diseases, dyskinesia, and hallucinosis. These days, studies on plant efficacy in traditional medicine are being conducted because herbal medicine is affordable, safe, and culturally acceptable and easily accessible. The Indian traditional medicine system called Ayurveda uses several herbs and medicinal plants to treat various disorders including neurological disorders. This review aims to summarize the data on the neuroprotective potential of the following common Indian spices widely used in Ayurveda: cumin (*Cuminum cyminum* (L.), Apiaceae), black cumin (*Nigella sativa* (L.), Ranunculaceae), black pepper (*Piper nigrum* (L.), Piperaceae), curry leaf tree (*Murraya koenigii* (L.), Spreng Rutaceae), fenugreek (*Trigonella foenum-graecum* (L.), Fabaceae), fennel (*Foeniculum vulgare* Mill, Apiaceae), cardamom (*Elettaria cardamomum* (L.) Maton, Zingiberaceae), cloves (*Syzygium aromaticum* (L.) Merr. & L.M.Perry, Myrtaceae), and coriander (*Coriandrum sativum* (L.), Apiaceae) in age-related neurological disorders.

## 1 Introduction

**FIGURE 1:**
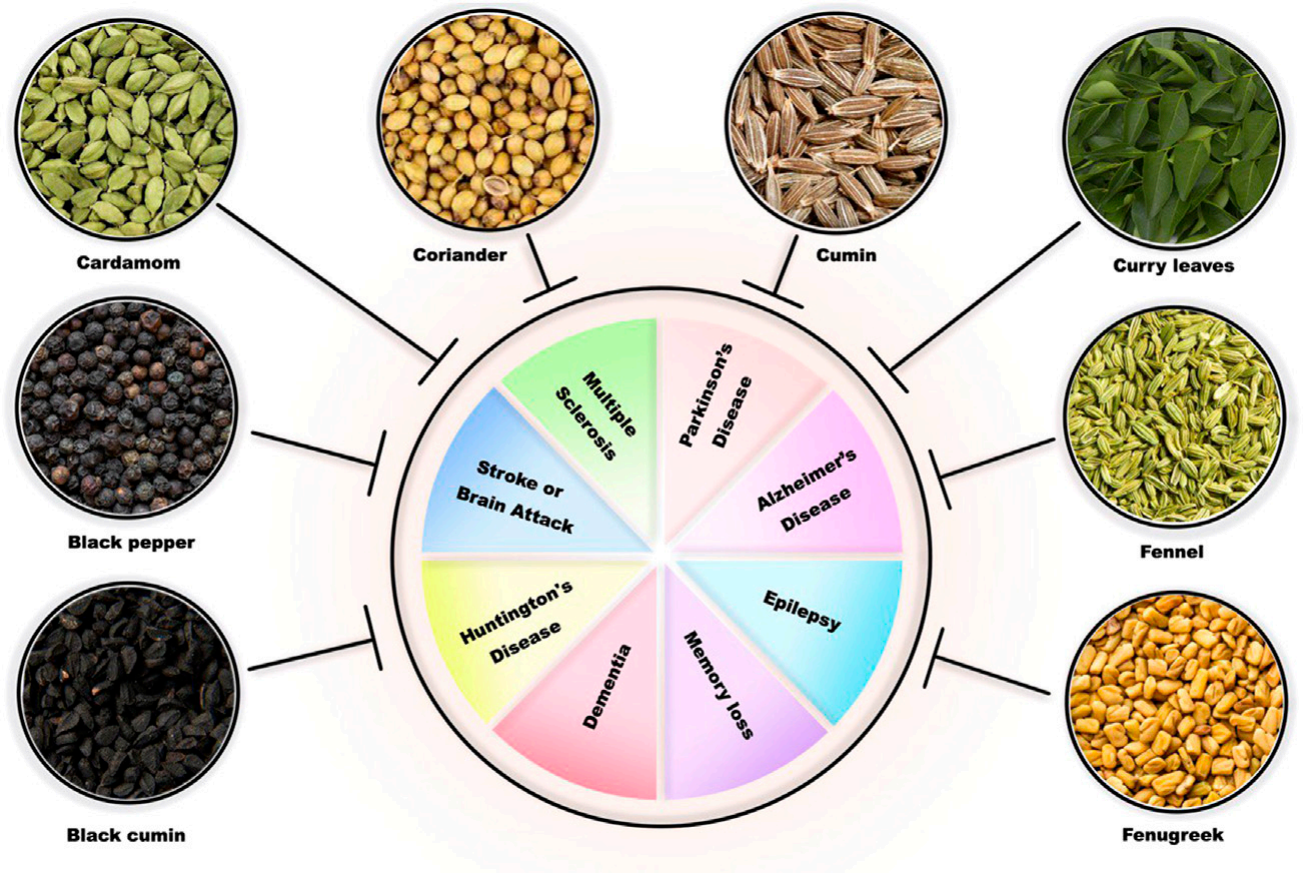
Potential of common Indian spices in ameliorating neurodegenerative diseases.

Aging is a complex natural biological process that involves damaging of several biochemical macromolecules, including proteins, DNA, and cellular organelles (mitochondria). Aging reduces cell proliferation by causing multiple shifts in biological processes such as energy metabolism, leading to cellular senescence. Aging gradually decreases biological functions of cells and increases the possibility of age-related disorders (Pyo et al., 2020). The prevalence of being diagnosed with multiple health conditions ranges from 55% to 98% in elders, referring to aging as a risk factor for multimorbidity (Marengoni et al., 2011). Aging is the main risk factor for several neurological disorders, including neurotoxicity, neuroinflammation, stroke, dementia, Alzheimer’s disease (AD), and Parkinson’s disease (PD) (Calne and Langston, 1983; Sulzer, 2007; Sparkman and Johnson, 2008; Mucke, 2009; Collier et al., 2011; Di Benedetto et al., 2017; Rao, 2018; Hou et al., 2019; Ijomone et al., 2020; Mecocci and Boccardi, 2020). Unfortunately, there are few or no efficient therapies for age-related neurodegenerative disorders, which tend to progress irreversibly and are associated with high socioeconomic and personal costs (Burns et al., 2006; Solfrizzi et al., 2017). In addition, the currently recommended treatment options for the aforementioned neurological disorders may lead to several side effects, such as nausea, diarrhea, gastrointestinal (GI) diseases, dyskinesia, hallucinosis, on–off phenomena, and possible loss of therapeutic effectiveness (Weiner et al., 1980; Casey et al., 2010). Due to the significant side effects of current treatments for age-related neurological diseases and the cost imposed on patients and the healthcare system, it is crucial to detect and develop more efficient approaches with less adversity toward these diseases. Regarding this issue, traditional medical treatments have gained the attention of many researchers due to the limited side effects and lower costs (Weiner et al., 1980; Casey et al., 2010; Singh et al., 2011; Liu et al., 2015; Pathak-Gandhi and Vaidya, 2017). Considering the main health issues in current society, including diabetes, arthritis, cardiovascular diseases, and cancer, the anti-proliferative, antidiabetic, anti-inflammatory, and anti-hypercholesterolemia effects of spices have maintained them an outstanding importance in the topic of traditional medicine. The low-calorie content of spices, rich source of antioxidants, and inexpensiveness make spices a desirable option for treatment (Vasanthi and Parameswari, 2010). Ayurveda as the traditional Indian medicine is considered to be one of the oldest medical systems, having a history spanning more than two millennia and medicinal plants being the main medicinal material (Jaiswal et al., 2016). Ayurveda carries a scientific tradition of harmonious living, which has been a traditional health system of Indian medicine since the old times (Mukherjee et al., 2017). That is why Ayurveda is referred to as “life science” (Mathpati et al., 2020). Various Ayurvedic medicines and remedies have been operated for the treatment of several diseases in humans (Mukherjee et al., 2017), including indigestion, common cold, chronic diarrhea, blood sugar, blood pressure (Ganguly, 2010), and especially age-related neurological disorders (Kannappan et al., 2011; Singh et al., 2011; Sedaghat et al., 2014; Pathak-Gandhi and Vaidya, 2017; Abulfadl et al., 2018). Common Indian spices including cumin (*Cuminum cyminum* (L.) [Apiaceae]), black cumin (*Nigella sativa* (L.) [Ranunculaceae]), black pepper (*Piper nigrum* (L.) [Piperaceae]), curry leaf tree (*Murraya koenigii* (L.) Spreng [Rutaceae]), fenugreek (*Trigonella foenum-graecum* L. [Fabaceae]), fennel (*Foeniculum vulgare* Mill [Apiaceae]), cardamom (*Elettaria cardamomum* (L.) Maton [Zingiberaceae]), cloves (*Syzygium aromaticum* (L.) Merr. & L.M.Perry [Myrtaceae]), and coriander (*Coriandrum sativum* (L.) [Apiaceae]) as part of the Ayurveda medication system are correlated with home remedies (Ganguly, 2010) and have been proven to impose great efficacy with regard to age-related neurological disorders (Youdim and Joseph, 2001; Adams et al., 2007; Chonpathompikunlert et al., 2010; Mani et al., 2013; Rasool et al., 2014; Morshedi et al., 2015; Prabha and Anusha, 2015; Morshedi and Nasouti, 2016; Nemati et al., 2018; Keshavarzi et al., 2019; Mima et al., 2020; Hannan et al., 2021; Zeng et al., 2021). Therefore, this review aims to summarize the data on the neuroprotective effect of these common Indian spices on age-related neurological diseases (Figure 1). Based on the search in the databases, 29 review studies were found. Our review article is more complete and comprehensive than all other review studies in this field. Until now, no study has been so complete on the effect of all these medicinal plants on age-related neurological disorders. On the other hand, these plants are in the group of Indian spices that are used in traditional Indian medicine, which is called the Ayurveda system. Ayurveda is an important science that is better to be used in modern medicine for treating various diseases, such as neurological disorders. This issue has been discussed in our review study, and this study is a guide for researchers to use these Indian plants in the treatment of neurological disorders.

## 2 Materials and methods

This study summarizes information about pharmaceutical potential of common Indian spices in age-related neurological disorders. In this article, the keywords searched include age-related neurological disorders, aging, neurotoxicity, stroke, dementia, Alzheimer’s disease, Parkinson’s disease, memory loss, neuroinflammation, migraine, epilepsy, brain tumor, multiple sclerosis, oxidative stress, reactive oxygen species, anti-atherogenic, atherosclerosis, cumin (*Cuminum cyminum* (L.) [Apiaceae]), black cumin (*Nigella sativa* (L.) [Ranunculaceae]), black pepper (*Piper nigrum* (L.) [Piperaceae]), curry leaves (*Murraya koenigii*), Spreng [Rutaceae]), fenugreek (*Trigonella foenum-graecum* L. [Fabaceae]), fennel (*Foeniculum vulgare* Mill [Apiaceae]), cardamom (*Elettaria cardamomum* (L.) Maton [Zingiberaceae]), cloves (*Syzygium aromaticum* (L.) Merr. & L.M.Perry [Myrtaceae]), and coriander (*Coriandrum sativum* (L.) [Apiaceae]). Searching for English articles published in journals until 2022 was performed in several databases, including PubMed, Scopus, and Google Scholar. Based on the search in the databases, 29 review studies were found. There was no book chapter in our search. As inclusion and exclusion criteria, we added all observational, *in vitro*, *in vivo*, and human studies to our article, which were related to the Indian spices mentioned previously in the article. A summary of the search strategy is included in Table 1.

**TABLE 1:** A summary of the search strategy in PubMed, Scopus, and Google scholar.

Search engine	Search strategy	Additional filter
Pubmed	((parkinson[Title/Abstract]) OR (alzheimer[Title/Abstract]) OR (epilepsy[Title/Abstract]) OR (multiple sclorosis[Title/Abstract]) OR (cerebrovascular disease[Title/Abstract]) OR (stroke[Title/Abstract]) OR (traumatic brain injury[Title/Abstract]) OR (huntigton[Title/Abstract]) OR (brain tumor[Title/Abstract])) AND ((Black cumin[Title/Abstract]) OR (Black pepper[Title/Abstract]) OR (Curry leaf tree[Title/Abstract]) OR (Fenugreek[Title/Abstract]) OR (Cumin[Title/Abstract]) OR (Elettaria cardamomum[Title/Abstract]) OR (Trigonella foenum graecum[Title/Abstract]) OR (Murraya koenigii[Title/Abstract]) OR (Pipper nigrum[Title/Abstract]) OR (Nigella sativa[Title/Abstract]))	English
120 articles were found		July23, 2022
Google scholar	1) Black cumin parkinson OR alzheimer OR epilepsy OR “multiple sclerosis” OR “cerebrovascular disease” OR stroke OR huntigton OR “brain tumor” OR “traumatic brain injury"	English
26 articles were found	2) Black pepper parkinson OR alzheimer OR epilepsy OR “multiple sclerosis” OR “cerebrovascular disease” OR stroke OR huntigton OR “brain tumor” OR “traumatic brain injury	July23, 2022
	3) Curry leaf tree parkinson OR alzheimer OR epilepsy OR “multiple sclerosis” OR “cerebrovascular disease” OR stroke OR huntigton OR “brain tumor” OR “traumatic brain injury"	
	4) Fenugreek parkinson OR alzheimer OR epilepsy OR “multiple sclerosis” OR “cerebrovascular disease” OR stroke OR huntigton OR “brain tumor” OR “traumatic brain injury"	
	5) Cumin parkinson OR alzheimer OR epilepsy OR “multiple sclerosis” OR “cerebrovascular disease” OR stroke OR huntigton OR “brain tumor” OR “traumatic brain injury"	
	6) " Nigella sativa” parkinson OR alzheimer OR epilepsy OR “multiple sclerosis” OR “cerebrovascular disease” OR stroke OR huntigton OR “brain tumor” OR “traumatic brain injury"	
	7) Piper nigrum parkinson OR alzheimer OR epilepsy OR “multiple sclerosis” OR “cerebrovascular disease” OR stroke OR huntigton OR “brain tumor” OR “traumatic brain injury"	
	8) Murraya koenigii parkinson OR alzheimer OR epilepsy OR “multiple sclerosis” OR “cerebrovascular disease” OR stroke OR huntigton OR “brain tumor” OR “traumatic brain injury"	
	9) Trigonella foenum graecum parkinson OR alzheimer OR epilepsy OR “multiple sclerosis” OR “cerebrovascular disease” OR stroke OR huntigto n OR “brain tumor” OR “traumatic brain injury"	
	10) Elettaria cardamomum parkinson OR alzheimer OR epilepsy OR “multiple sclerosis” OR “cerebrovascular disease” OR stroke OR huntigton OR “brain tumor” OR “traumatic brain injury"	
Scopus, 98 articles were found	(TITLE-ABS-KEY(Parkinson)OR TITLE-ABS-KEY(Alzheimer)OR TITLE-ABS-KEY(Epilepsy) OR TITLE-ABS-KEY(“Multiple sclerosis”) OR TITLE-ABS-KEY (Stroke) OR TITLE-ABS-KEY(” traumatic brain injury”) 0R TITLE-ABS-KEY(Huntington)OR TITLE-ABS-KEY(“brain tumor”) AND (TITLE-ABS-KEY(“Black cumin”)OR TITLE-ABS-KEY (“Black pepper”)OR TITLE-ABS-KEY(“Curry leaf tree”)OR TITLE-ABS-KEY(Fenugreek)OR TITLE-ABS-KEY(Cumin) OR TITLE-ABS-KEY(“Elettaria cardamomum”) OR TITLE-ABS-KEY(“Trigonella foenum graecum”)OR TITLE-ABS-KEY(“Murraya koenigii”) OR TITLE-ABS-KEY (“Piper nigrum”) OR TITLE-ABS-KEY (“Nigella sativa”)OR TITLE-ABS-KEY (“Murraya koenigii”)	English
		July23, 2022

JBI’ critical appraisal checklist was used to assess the quality of included articles (https://jbi.global/critical-appraisal-tools).

## 3 Definitions

### 3.1 Parkinson’s disease

In the early 18th century, Dr. James Parkinson described Parkinson’s disease (PD) as “shaking palsy.” This is a chronic progressive neurodegenerative disorder, which affects both motor and non-motor functions, mobility, and muscle control (DeMaagd and Philip, 2015). In patients with PD, the impairment of dopaminergic neurons in the substantia nigra pars compacta leads to the loss of dopaminergic function. When dopamine is lost from the striatum, the globus pallidus segment/reticulate portion of the substantia nigra circuit hypreactivates, which results in the dysfunction of gamma aminobutyric acid and inhibition of thalamo motor activity (Beaulieu and Gainetdinov, 2011).

Pathological changes and behavioral symptoms similar to those in PD patients are replicable by certain neurotoxins such as rotenone (Fathalla et al., 2016). In fact, rotenone inhibits mitochondrial electron transport chain complex I, leading to death of dopaminergic neurons (Hirsch and Hunot, 2009). This inhibition also upregulates generation of free radicals followed by microglia activation. Activated microglias are involved in translocation of redox-sensitive nuclear factor kappa-B to the nuclear environment that subsequently causes TH enzyme loss (Sawada et al., 2006). This loss, besides death of dopaminergic neurons, result in motor deficits (Dhanalakshmi et al., 2016). Moreover, rotenone promotes hypolocomotion and loss of rearing activity in rodent models (Fathalla et al., 2016). Distortions of cytoarchitecture and elimination of neuronal cells are seen in the substantia nigra and striatum of rats treated with rotenone (Villalba et al., 2009; Sharma and Nehru, 2013). Severe dropping of dendritic spines and decreased dendritic length of the striatum in PD brain are also documented in rotenone-treated rats (Stephens et al., 2005; Zaja-Milatovic et al., 2005).

A number of factors contribute to the development of PD, including, environmental changes, aging, chronic diseases, and social disadvantage (Schrag et al., 2015). A strong relationship between PD and aging has been identified for several decades. As with AD, aging is the main risk factor for the extension and development of PD. The PD prevalence increases about 10 times between the age of 50 and 80 (Collier et al., 2011). The logic behind these statics probably attributes to age-related changes, which also makes patients prone to PD development. This includes mitochondrial instability (Przedborski and Jackson-Lewis, 1998), increase of blood–brain barrier permeability (Elahy et al., 2015), inflammation of dopaminergic neurons (Nagatsu et al., 2000; Verma et al., 2014), reduction of antioxidant system function, and increase of free radical production. PD treatment includes a wide range of medico-pharmacological therapies and revolutionary surgical interventions such as deep brain stimulation (DBS), which has been proposed in recent years. However, there is still no definitive therapy that modifies the disease (Beitz, 2014). There are also concerning side effects for some PD treatment methods including dyskinesia, hallucination, on-off phenomena, and possible loss of therapeutic effectiveness, which emphasizes on the need for more efficient drugs with a better safety profile (Weiner et al., 1980).

### 3.2 Alzheimer’s disease and dementia

Alzheimer’s disease (AD) is an age-related neurologic disorder with characteristics of amyloid (Aβ) plaques and neurofibrillary tangle accumulation, as well as significant neuronal deterioration and synaptic disruption. Brain shrinkage (atrophy) and brain cell death are found to be the results of these changes (Mucke, 2009; Teymuori et al., 2021). AD is known to be the most prevalent form of dementia in which memory deficit could not be recovered (Teymuori et al., 2021). Dementia itself is defined by severe and progressive decline of cognitive status, particularly the memory function, which may lead to complete loss of daily abilities of the patient. This condition is believed to be strongly linked with the aging process, since its prevalence among individuals over 85 years makes up to almost 50 percent and nearly 10 percent of those aged 65 years or above may develop some form of memory impairment related to dementia (Dorszewska et al., 2016).

The majority of AD cases is sporadic with the underlying causes remain to be fully elucidated. Thereby, genetic mutations associated with familial AD are used to establish animal models since downstream events are quite similar between two types (LaFerla and Green, 2012). Moreover, not all aspects of the disease are existed in a single mouse model, and only one or two components of AD could be analyzed in each model. For instance, mutant mice with APP overproduction show similar pathology to that of human brain, that is, extracellular plaques with accumulated β-amyloid (Findeis, 2007). Similar to the human brain, animal plaques are stained with both thioflavin and Congo red, and they show similar fibrillary structures. Noteworthy, toxicity of oligomeric Aβ and its central role in AD were evidenced by the use of transgenic mice. However, there are also some inconsistencies between animal models and human brain including, but not limited to, cognitive decline and intraneuronal aggregates of hyperphosphorylated tau (LaFerla and Green, 2012).

As mentioned, AD is known to be the most common cause of dementia (Prendecki, 2016). However, other neurodegenerative disorders, such as Parkinson’s, may present with dementia in terminal stages (Coelho and Ferreira, 2012). In the course of AD and also during the natural aging process, the adverse changes of neural cells and synapses in regards to integrity and function can lead to memory problems (McKhann et al., 2011). The degeneration process in both AD and aging is accelerated by the activation of glial cells and subsequent overproduction of pro-inflammatory molecules (McGeer and McGeer, 2001). More importantly, it is highly speculated that cholinergic neurons, which express acetylcholine receptors and are mainly based in the basal forebrain, are more susceptible to damage as AD or aging itself advances (Parsons et al., 2013; Reeve et al., 2014). The loss of these types of cells results in a significant decrease in the function of cholinergic synapses followed by the reduced release of Ach in the brain, which, based on the “cholinergic deficit hypothesis”, is the logic behind cognitive dysfunction and behavioral disabilities in AD patients and those with advanced age (M Tata et al., 2014). Furthermore, the role of oxidative stress in inducing neurological damage and memory loss should not be overlooked, as it has been found to have a reciprocal relation with AB aggregation (Yatin et al., 1999; Butterfield and Lauderback, 2002; Cioanca et al., 2013). Therefore, the therapeutic approaches against dementia, whether as a symptom of AD and similar disorders or as a result of natural aging, are based on inhibiting the acetylcholinesterase enzyme or ameliorating the neuroinflammatory process and oxidative stress (Knopman, 2006; Liu et al., 2007; Weggen et al., 2007). Studies investigating the effects of natural products derivatives on dementia and AD are also mainly focused on the ability of these compounds to reverse the cholinergic deficiency, oxidative stress, and inflammatory changes.

Recent advances in neuroscience have revealed a greater, in-depth understanding of the complexities associated with memory. Contemporary theories hold that an integral relationship between memory formation, stabilization, and consolidation revolve around plasticity of neuronal networks. The associated requisite receptors α-amino-3-hydroxy-5-methyl-4-isoxazole propionic acid (AMPA) and N-methyl-D-aspartate (NMDA) and cellular mechanisms surrounding plasticity (posed to incite molecular functionality) also display strong correlations in the pathogenesis of dementias. When the brain is in a diseased state as a result of malignant neurotransmission (i.e., in Alzheimer’s disease; AD), the homeostatic balance required for normal neuronal processes is disrupted, which leads to degeneration of neural circuitry. Present efforts to find new treatments aimed at reversing or halting neurodegeneration are immense, with increasing attention being placed on investigating various herbal medicines. A wide variety of herbal plants (i.e., *Panax ginseng*, *Polygala tenuifolia*, *Acorus gramineus*, and *Huperzia serrata*, examined here within), extracts, and compounds have, to date, already presented advantageous results when tested against known pathogenic markers related to AD-associated dementia. The efficaciousness of herbal medicines appears to be a modulatory effect on neurotrophins, kinases, and their substrates that, in turn, initiate or take part in intracellular cascades related to memory processes (Jesky and Hailong, 2011).

### 3.3 Epilepsy

Epilepsy is a central nervous system condition with a higher incidence rate in children and elderlies. Individuals with epilepsy often suffer from physical problems such as bony fractures and tend to have higher rates of mental disorders. Epilepsy is characterized by experiencing recurrent unprovoked seizures. Symptoms might vary depending on the area of the brain that is involved. Loss of consciousness, impairment in cognitive function and movement disturbance are among common symptoms. Abnormal activity of cortical neurons is responsible for initiating seizure and axons and glial cells might be involved. Epileptogenic networks are distributed widely in generalized epilepsy and can involve thalamocortical structures. In focal epilepsy, disruption in neural networks is usually in one hemisphere (Thijs et al., 2019).

Epileptogenesis is defined as the cellular and structural mechanism that transforms a normal brain into an epileptic one after a transient insult (Pitkänen et al., 2015). Rodent models of temporal lobe epilepsy (TLE) are considered as suitable tools for studying early stages of epileptogenesis, since human hippocampal tissue is almost unavailable for this aim. Chemoconvulsants such as kainic acid and pilocarpine are used to establish human hippocampal sclerosis with distinguished neuropathological features such as recurrent chronic seizures (Leite et al., 2002). Kainic acid is an L-glutamate analog that induces neuron depolarization and epileptic attacks in rodents with a remarkable injury within hippocampal formation (Sharma et al., 2007). When kainic acid and pilocarpine are administered systematically, damage is extended to neocortical regions (Sharma et al., 2007) reflecting the patterns of human TLE. Pilocarpine-induced lesions in animal models lead to spontaneous recurrent seizures (Cavalheiro et al., 1991) with interictal activity patterns as same as human hippocampal tissue (Knopp et al., 2008)

Epilepsy is among the most common neurological conditions in older people. Its intermittent and unpredictable nature makes epilepsy more important for this group of people. Certain processes such as the effect of age on the risk of recurrent convulsive excitoxic status epilepticus are governed by sophisticated mechanisms, which necessitates the need for further investigations in preclinical animal models (Becker and neurobiology, 2018)

Physical injury is more frequent in the elderly because of the increased tendency for falls. Neurodegenerative conditions, head injuries, and brain tumors are found to be the most known causes of epilepsy in older patients with epilepsy (Johnston and Smith, 2010).

### 3.4 Multiple sclerosis

Multiple sclerosis, a chronic neurological disorder, causes demyelination of the nerves in the brain and spinal cord (Kutzelnigg and Lassmann, 2014). The possible link between MS and aging has been the topic of various studies. It is evident that as individuals age the function of repair system declines, explaining the accelerated progress of disease in aging patients with a history of MS (Rist and Franklin, 2008). Also, aging contributes to the increased iron deposition in CNS due to the elevated expression of the ferritin protein in oligodendrocytes and microglia. This phenomenon matters since the iron deposition promotes oxidative stress and apoptosis and eventual worsening of disease (Andersen et al., 2014). MS can also be considered an autoimmune disorder because in the course of disease, T cells attack CNS autoantigens in genetically predisposed people (Polman et al., 2011) With aging, immune system imbalance is more occurring; hence, cells including neurons are exposed to a chronic inflammatory state (Cevenini et al., 2013).

Multiple sclerosis (MS) is a demyelinating disease affecting the central nervous system, with no curative medicine available. The use of herbal drugs and dietary supplements is increasing among people with MS (PwMS), raising a need for knowledge about potential interactions between conventional MS medicine and herbal drugs/dietary supplements. This systematic review provides information about the safety of simultaneous use of conventional MS drugs and herbal drugs frequently used by PwMS. The study included 14 selected disease-modifying treatments and drugs frequently used for symptom-alleviation. A total of 129 published studies found *via* PubMed and Web of Science were reviewed according to defined inclusion and exclusion criteria. Findings suggested that daily-recommended doses of *Panax ginseng* and *Ginkgo biloba* should not be exceeded, and herbal preparations differing from standardized products should be avoided, especially when combined with anticoagulants or substrates of certain cytochrome P450 isoforms. Further studies are required regarding ginseng’s ability to increase aspirin bioavailability. Combinations between chronic cannabis use and selective serotonin reuptake inhibitors or non-steroidal anti-inflammatory drugs should be carefully monitored, whereas no significant evidence for drug interactions between conventional MS drugs and ginger, cranberry, vitamin D, fatty acids, turmeric, probiotics, or glucosamine was found (Petersen et al., 2021).

### 3.5 Cerebrovascular disorders

The term “cerebrovascular disease” refers to any condition in which the blood supply of the brain is hindered due to vessel(s) involvement including stenosis, thrombosis, embolism, and hemorrhage (Khaku, 2021). This results in insufficient cerebral blood flow cognitive and physical disabilities, which also impose a significant socioeconomic burden. For instance, stroke as the most common type of cerebrovascular disease accounts for five million deaths annually, and each year more than 116 million years of healthy life is lost to death and disability caused by stroke (Lindsay et al., 2019). It is worth noting that the incidence of cerebrovascular disorders increases with aging, thus age might play a prominent role in developing vascular events (Hollander et al., 2003). As individuals age, the cells gradually enter to a state called cellular senescence in which the cell division is permanently abstained and the pattern of gene expression and normal cell function is altered (Bianchi, 2007). Subsequently, these cells release pro-inflammatory cytokines such as IL-1, IL-6, and IL-8 in an autocrine or paracrine manner that accelerate the senescence of neighbor cells (Hubackova et al., 2012; Pantsulaia et al., 2016). Therefore, aging can be interpreted as a mild but chronic inflammation. This process is also inevitable in endothelial cells, functioning in axes such as regulating metabolism and preserving blood flow (Aird, 2012). Ischemia/reperfusion (I/R) injury as a prominent damaging mechanism of stroke also might get worsen in elders due to aging and the augmenting factor for adverse changes attributed to I/R, including oxidant/antioxidant imbalance, excitotoxicity, dysregulated inflammatory responses, mitochondrial dysfunction, and apoptosis (Li et al., 2018). Also, it is evident that forming atherosclerotic plaques, a major risk for cerebrovascular diseases, is associated with the functional disturbance in senescent endothelial cells (Pantsulaia et al., 2016).

Herbal drugs are regarded to be effective in stroke treatment. Herbs have fewer recorded side effects than allopathic medicine and may be safer to take for a prolonged period of time. Herbal drugs are regarded to be more effective in treating chronic disorders.

A stroke is an example of a health problem. Several therapeutic plants and their active ingredients components show up in laboratory investigations.

The difficulties in adapting laboratory animal findings into clinical trials, on the other hand, have created a substantial barrier to the use of herbal treatment in stroke. Efforts should be made to continue using tried-and-true treatment alternatives until scientifically rigorous confirmation of the efficacy and safety of herbal medicine in ischemic stroke patients is produced. Natural compounds should be given more thought, since they can have extended therapeutic time windows, good pharmacological targets, and little side effects. Herbal medicine has a bright future in the treatment of ischemic stroke, but more effort is needed to convert animal study results to human use. Natural compounds derived from traditional medicinal plants have received increased attention in the field of drug development as a possible treatment alternative for cerebral ischemia with low systemic side effects that might restrict their long-term usage (Ghosh et al., 2014; Gaire, 2018).

### 3.6 Traumatic brain injury

Traumatic brain injury (TBI) is a brain injury acquired from an external force. This event causes death and long-term disability in traumatic patients. The severe forms of TBI involve the brain entirely and exhibit extensive injury along with swelling. The outcome of brain injuries presents in different forms ranging from mild consciousness alteration to comatose state and death. (Galgano et al., 2017). According to epidemiological studies among patients with head injuries, older patients are more prone to intra-cranial bleeding followed by a traumatic injury than younger patients (Vollmer et al., 1991; Coronado et al., 2005). Statics indicate a higher rate of hospitalization and mortality followed by TBI in adults with age of 75 and above compared to younger counterparts (Centers for Disease Control and Prevention). Falls are considered the major cause of TBI for older adults, with motor vehicle traffic crashes being the second (Langlois et al., 2006). To determine the reason for the severity of complications in elderlies, several aspects of age-related changes should be considered. As individuals age, the adherence of dura matter layer to the skull increases. In addition, patients usually are under treatment with anticoagulant agents, which predisposes them to bleeding injuries. As mentioned previously, cerebrovascular atherosclerosis and increased ROS generation occurs with aging, which increases the risk of vascular damage and post-TBI oxidative stress (Timiras, 2002; Thompson and Bourbonniere, 2006).

Traditional treatment options for mild traumatic brain injury (TBI) have had only little therapeutic effectiveness. Treatment for mild TBI is becoming increasingly popular due to its link to the development of chronic traumatic encephalopathy and other neurodegenerative illnesses, although therapeutic choices remain limited. Traditional pharmacological approaches for the treatment of mild TBI have failed to make the leap to the clinic. As a result, numerous pre-clinical research works are currently focusing on non-pharmaceutical treatments for TBI. Treatment options are difficult to implement due to the complexities of its repercussions. Given its complexities, TBI is a unique target for complementary and alternative medicine (CAM) therapies (Hernández et al., 2016; Lucke-Wold et al., 2018).

### 3.7 Huntington’s disease

Huntington’s disease (HD) is a progressive, autosomal-dominant, and fetal neurodegenerative disease caused by abnormal expansion of CAG repeat in the Huntington gene. Dystonia and choreatic movements, incoordination, depression, and cognitive deficits are HD symptoms. Chorea is the most noticeable characterization and responds clearly to medication. Symptoms usually begin between ages 20–65 years, and there is a negative relationship between age at disease onset and CAG repeats number. HD is a disease with limited management options, and also many efforts are made to use silencing techniques; there is still a long way to pave (Wyant et al., 2017). Aging is known as a risk factor for neurodegenerative diseases. Biological alterations in aging include mitochondrial dysfunction, telomere attrition, epigenetic changes, genomic instability, intracellular communicating alteration, and cellular senescence. Many of aging hallmarks also play a role in HD pathogenesis (Machiela and Southwell, 2020).

A variety of transgenic, knock-in, and conditional mouse models of HD have been produced including R6/1, R6/2 (Mangiarini et al., 1996), YAC72, YAC128 (Hodgson et al., 1999), Tg100 (Laforet et al., 2001), CAG71, and CAG94 (Kumar et al., 2010). As an example, genetically manipulated models of HD with mouse htt expression provide a unique opportunity for studying evolution of pathogenic processes. It is obvious that disease phenotype is associated with the type of incorporated mutant gene. Models that express N-terminal fragments of HD exon 1 shows a rapid development of disease phenotype accompanied by motor deficits and weight loss seen in HD patients, whereas those models that contain the full-length murine Hdh gene demonstrate a lengthy disease course with attenuated motor defects and albeit specific neurodegeneration signs. Another strategy to establish animal models of HD is using pharmacologic inhibitors of mitochondrial complex II. Decreasing the activity of complex II in respiratory chain of the affected brain regions causes striatal damage and motor phenotypes that are closely similar to symptomatic HD patients. In this regard, mitochondrial toxins such as 3-NP and malonate, besides excitotoxins such as quinolinic acid, induce oxidative damage with HD-like lesions (Kumar et al., 2010).

### 3.8 Brain tumor

A brain tumor is characterized by the overgrowth of cells in the brain or the central spinal canal. There is an increasing incidence of brain tumor in the population aged 65 years or over (Wrensch et al., 2002). Despite the biological link between aging and brain tumors remaining obscure, lessons can be learned from the effects of natural products on other types of cancers. A total of four main strategies are deployed to establish brain model malignancies in mice. These include chemical mutagen-induced, xenograft transplantation, germline genetic modification, and somatic genetic modification (Dai and Holland, 2001). The latter three are the mostly used methods. To study the efficacy of novel drugs and seek gene functions, xenograft models are used in preclinical trials. In this regard, glioma cell lines are injected subcutaneously or into the brain of immunocompromised mice (Kaye et al., 1986). The U251 glioma model is one of the models that is similar to human glioblastoma with regard to infiltrative invasion into the brain parenchyma; considerable foci of necrosis, positive markers of GFAP, S100B, and vimentin (Jacobs et al., 2011); losses of tumor suppressor genes (p53 and PTEN); and deletion of INK4a/ARF (Radaelli et al., 2009). However, other models may demonstrate different appearance. For example, U87 shows a non-diffusely infiltration with a well-defined tumor border and rare necrotic foci (Candolfi et al., 2007; Radaelli et al., 2009). Genetically engineered models (germline modification and somatic cell gene transfer) are generated by molecular manipulation. DNA is combined into totipotent cells before the developmental stage, whereas in somatic gene transfer, the model is nonhereditary and a particular subset of cell population is targeted (Chen et al., 2013).

## 4 Black cumin (*Nigella sativa* (L.) [Ranunculaceae])

Black cumin is a plant naturally growing in East Asia and Mediterranean (Norman, 1991). It has been used as a medicinal herb for centuries due to the high potential of its active components, especially thymoquinone (TQ), in treating multiple chronic ailments, including neurological diseases (Kooti et al., 2016). Other constituents are known to be phenolic compounds, alkaloids, sterols and saponins, lipid-based components, and volatile oils consisting of several compounds (Botnick et al., 2012). Impressive outcomes and the minimal side-effect profile have drawn the attention of many researchers to explore its effects on various neurological disorders.

### 4.1 Parkinson’s disease

In patients suffering from neurological disorders including PD, synapse degeneration is common. According to Two separate studies on the neuroprotective effects of TQ against synaptic toxicity, TQ can improve PD-induced changes in synapses of hippocampus and related symptoms. To be more specific, the decrement in the expression of synaptophysin and the synaptic vesicle recycling increment induced by alpha-synuclein can be attenuated by TQ, hence protects against synaptic toxicity in primary hippocampal and hiPSC neurons of rats. It was shown that TQ (100 nM) (1 g/ml FM1-43 for 5 min(, can have inhibitory effects against the synaptic activity, protective effects on cultured hippocampal neurons against synapse damage induced by alpha SN, and decrement effects on synaptophysin level (Alhebshi et al., 2014; Elibol et al., 2019). Furthermore, TQ restored synaptic vesicle recycling inhibition induced by mutated P123H in hippocampal neurons and therefore protected human hiPSC neurons against the spontaneous firing activity inhibition (Alhebshi et al., 2014). Ardah et al., reported that the oxidative stress and inflammatory response induced by MPTP (25 mg/kg b. wt.) in PD mice models can be protected by TQ (Ardah et al., 2019). Since it was TQ (10 mg/kg body weight [b. wt.]) was administered for 1 week prior to MPTP) also reduced the elevated levels of pro-inflammation mediators including COX-2 and iNOS. In case of PD induced by other toxins such as rotenone, TQ was also able to minimize the impairments regarding motor function, and the level of dopamine, parkin, tyrosine hydroxylase (TH), and dynamin-related protein 1 (Drp1) in affected rats. According to Fattah et al. (Fattah et al., 2016) Movement assessing tests including movement failure, bar test, and rearing test as well as pre-albumin serum level and antioxidative profile were also improved when TQ was given in doses of 7.5 and 15 mg/kg PO. Similarly, Dong et al. (2021) proposed antioxidant and anti-apoptotic properties of TQ (0.25–2.0 μM for 24 h), explained by the process of up-regulating Nrf2/ARB signaling pathway and subsequent increase in Antioxidant enzymes expression such as glutathione-S-transferase (GST), quinone oxidoreductase (NQO1), and Heme oxygenase 1 (HO-1). The possibility of this mechanism is highlighted by the fact that addition of siRNA (suppressor of Nrf2) reversed the TQ protective effects on nigrostriatal dopaminergic neurons. In another study, Radad et al. (2015) found that TQ (0.01, 0.1, 1, and 10 μM) can inhibit apoptotic cell death by preserving mitochondrial membrane potential, activating lysosomal degradation and reducing the LDH release. This chain of events protects dopaminergic neurons of mesencephalic, against cell death and toxicity induced by 1-methyl-4-phenylpyridinium (MPP+). (Radad et al., 2015). The findings of this study was in line with the results of Dowlati et al. (2010). Also, Pretreatment with TQ (5 and 10 mg/kg) is suggested by Sedaghat et al. (2014), since it significantly improved turning behavior, decreased the level of malondialdehyde (MDA), and prevented the neuronal loss of substantia nigra pars compacta (SNC). Therefore, it can be concluded that TQ as a major component of black cumin can be used as a promising addition to conventional drugs prescribed for PD.

Other components of black cumin in different preparations can exert different efficacy and effectiveness. In a study by Sandhu and Rana (2013), the anti-Parkinson activity of ethanolic extract of Nigella sativa L. (EENS) in case of neurotoxicity induced by chlorpromazine (CPZ) in animal models was shown. The ethanolic extract remarkably suppressed the induced catalepsy at doses of 200 and 400 mg/kg when compared to Chlorpromazine treated group. The lipid peroxidation index and the raise in nitrite level were remarkably reversed when ethanolic extract of Nigella sativa (*N. sativa*) (200 and 400 mg/kg) was used. It was also observed that the level of GSH was remarkably increased when the same amount of the ethanolic extract was applied (Sandhu and Rana, 2013).

In another study by Jahromy et al. (2014), the effect of orally used hydroalcoholic extract of *N. sativa* on muscle stiffness in muscle rigidity induced by perphenazine was assessed. *N. sativa* (100 mg/kg, 200 mg/kg) remarkably improved the score of muscle rigidity in a dose-dependent manner (with 100 mg/kg starting at the 40th minute and all times with 200 mg/kg) compared to control group who received only water.

In a rat model study, Baluchnejadmojarad et al. (2014) investigated the effect of Carvacrol (CAR) ((10 mg/kg) started 3 days before the surgery). As a monoterpenic phenol compound of *N. sativa* on Parkinson’s disease induced by unilateral intrastriatal 6-hydroxydopamine. Rotational behavior (indicator of nigrostriatal dopamine degeneration) (Shapiro et al., 1987), and the concentration of stress oxidative markers in the midbrain, was measured 2 weeks after Carvacrol administration: it is reported that carvacrol inhibited the production of ROS and lipid peroxidation. Also, lesioned rats given carvacrol showed decrease in rotation number, decreased levels of nitrite oxide and the MDA, as well as increased antioxidant enzyme catalase.

Moreover, A study by Hosseinzadeh et al. (2017) was performed to investigate the neuroprotective effect of fatty acids (FA) of *N. satvia* against the neurotoxicity induced by MPP+ ((1.5 mM) for 24 h). They found that fatty acids partially attenuated apoptosis mediated by MPP + through caspase-3 and -9 activity inhibition and increasing the mitochondrial membrane potential (MMP). Also, a mixture of oleic acid, palmitic acid, and linoleic acid decreased the MPP^+^-induced COX activity in PC12 cells. by considering the anti-inflammation and anti-apoptotic effects of *N. sativa* fatty acids, these fractions may lead to neuroprotective effects and thus can be added to dietary supplements.

### 4.2 Memory loss

*N. sativa* effects on memory have been subject to only a few studies, but existing evidence point out that it would have a positive impact on memory and learning (Alasmari et al., 2022; Kantar et al., 2022; Khan et al., 2022; Xue et al., 2022). In the radial arm maze test of rats, research has shown that both long- and short-term administration of *N. sativa* oil can improve working memory, especially short-term memory. According to Sahak et al. (2013) In both the T-maze alternation task and the object recognition test, *N. sativa* oil with (6.0  μl/100 g body weight of Nigella sativa oil for 20 weeks) restores spatial and non-spatial working memory function. A study by Perveen et al. (2008) showed that *N. sativa* increases 5-HT levels in the brain in a similar manner to anxiolytic drugs. Also, Sayeed et al. (2013) concluded that the seeds of *N. sativa* (two 500 mg capsules once daily after dinner for 9 weeks), may have mild memory enhancement properties in elderly people by preventing the destruction of acetylcholine and inhibiting further neurodegeneration. Based on Sahak et al.’s (2013) findings, *N. sativa* oil not only has been shown to inhibit enzymes such as acetylcholinesterase (AChE), it also affects brain tumor necrosis factor-alpha and glutathione, so it might be a promising neuroprotective agent. Also, Bhandari (2014) observed that *N. sativa* hydro-alcoholic extract prevents scopolamine-induced spatial memory deficits, decreases anticholinesterase activity in brain tissue, and reduces oxidative stress. As a result, *N sativa* causes an increase in aspartate and glutamate levels, while GABA and glycine levels are decreased.

### 4.3 Multiple sclerosis

The therapeutic effects of extracts of *N. sativa* against Multiple Sclerosis were reported by Noor et al. (2015) in an animal model study. *N. sativa* (2.8 g/kg body weight) reduced inflammation processes, enhanced and increased remyelination in CNS, and suppressed TGF β1 expression in experimental autoimmune encephalomyelitis (EAE) models of Multiple Sclerosis disease.

In a study by Mohamed et al. (2009), remarkable protection (90%) of TQ extract of *N. sativa* against EAE was observed in rats. Therefore, TQ might prevent the chronic relapsing phase of multiple sclerosis. *N. sativa* also prevented ROS production, followed by the decrease of NO and MDA levels in both brain and medulla spinal tissues. Oxidative stress is implicated in the onset and progression of EAE in an experimental model. In this regard, reducing oxidative stress could alleviate EAE signs and symptoms. Mohamed et al. (2003) found that TQ (1 mg/kg) was injected into the tail vein of female Lewis rats after EAE was induced using myelin basic protein emulsified with complete Freund’s adjuvant. In EAE animals, TQ has been observed to inhibit oxidative stress. Combined TQ with myelin basic protein treatment prevented and ameliorated EAE symptoms. Mohamed et al. (2005) reported that TQ (1 mg/kg per day for 10 days) was effective in blocking perivascular cuffing and mononuclear cell infiltration in the brain and spinal cord, enhancing red blood cell glutathione (GSH), and inhibiting NF-ĸB activation in the brain and spinal cord.

### 4.4 Stroke

There is evidence that black cumin and TQ can be useful in preventing strokes and traumatic brain injuries. Multiple studies proposed an anti-inflammation and anti-oxidative mechanism to explain the improvements attributed to TQ (Al-Majed et al., 2006; Hosseinzadeh et al., 2007a; Hosseinzadeh et al., 2007b; Hajzadeh et al., 2011). Inhibiting lipid peroxidation, decreasing MDA levels in the brain, downregulating the iNOS expression, and subsequent decrease in NO production, as well as the rise in the activity of antioxidant enzymes (SOD, GSH, CAT) are found to be responsible for the benefits of pre-treatment and treatment with TQ. It is reported that due to these changes, TQ oral administration in different doses (2.5, 5, and 10 mg/kg) and regimens (isolated or combined with other forms of *N. sativa*) results in less hippocampal neural damage and neuroprotection throughout the ischemic and reperfusion period (Baluchnejadmojarad and Roghani, 2013). Also, according to Gökce et al. (2016) the same mechanism could explain the inhibition of IRI-induced apoptosis of motor neurons and spinal cord tissue preservation with TQ pre-treatment (10 mg/kg of TQ intraperitoneally for 7 days before induction of spinal cord I/R injury, and administration was continued until the animal was euthanized). It is worth noting that prophylactic TQ might be helpful to prevent stroke as Guan et al., found that TQ can improve blood pressure, cognition deficits, and ameliorated memory in rats with spontaneous hypertension prone to stroke (Guan et al., 2018). Furthermore, few studies aimed to enhance the mentioned properties by suggesting a different administration route for TQ. Ahmad et al. (2016) showed that noninvasive intranasal administration of TQ improves neurobehavioral activity (locomotor and grip strength) in cerebral ischemia induced by occlusion of the middle cerebral artery. Also, Xiao et al. (2016) found that PLGA-chitosan nanoparticles loaded with TQ (quivalent to 5 mg/kg/day TQ) intranasally for 12 days), show optimal nasal-to-brain transmission and neuroprotective effect against cerebral ischemia.

Moreover, several studies investigated the potential of *N. sativa* preparations and its components in stroke treatment. In a study by Akhtar et al. (2012), it was found that *N. sativa* may elevate reduced GSH, catalase levels, and superoxide dismutase (SOD) by two of its compounds named hydro-alcoholic and aqueous extracts. Therefore, mentioned anti-ischemic effects attributed to antioxidant properties and free radical scavenging caused by *N. sativa*. Also, in another study by Akhtar et al. (Akhtar et al., 2013), it was found that petroleum ether and chloroform extracts of *N. sativa* (400 mg/kg for 7 days) can have protective effects in cerebral ischemia by improving motor activity and gaining strength, which again emphasize on the anti-inflammatory and antioxidant effects of *N. sativa* since, both mentioned extracts elevated the levels of glutathione, SOD, and catalase (Akhtar et al., 2013). In an animal study by Hobbenaghi et al. (2014), it was shown that *N. sativa* (1 mg/kg, 10 mg/kg, 50 mg/kg). Extract can prevent edema and support neuronal tissue of the hippocampus. Soleimannejad et al. (2017) showed that black cumin seed extract improved global ischemia outcomes at doses of 10 and 20 mg/kg through an angiogenic process involving up-regulating vascular endothelial growth factor (VEGF) gene expression, matrix metallopeptidase (MMP9), and hypoxia-inducible factor-1 (HIF). Taken together, black cumin could be considered as an acceptable substance for the treatment and prevention of cerebral ischemic/reperfusion.

### 4.5 Traumatic brain injury

A study conducted by Kamasak et al. (2021) showed that *N. sativa* (400 mg/kg) exhibits neuroprotective effects on mice affected with brain trauma. It was found that total antioxidant status and cell viability was higher in the group that *N. sativa* was administered 1 day prior to trauma. Thus, indicating *N. sativa* potency in preventing apoptosis in the presence of brain injury as well as reducing oxidative stress and lipid peroxidation. In addition, oral administration of TQ resulted in reduced lactate dehydrogenase (LDH) activity, MDA levels in neurons nuclei and mi in brains of rats with TBI (محمد, 2017). TQ (5 mg/kg/day for 7 days) also exhibits neuroprotective and ameliorating effects by improving the neural density in injured area and decreasing MDA levels in neurons nuclei of TBI rat models (Gülşen et al., 2016).

**FIGURE 3:**
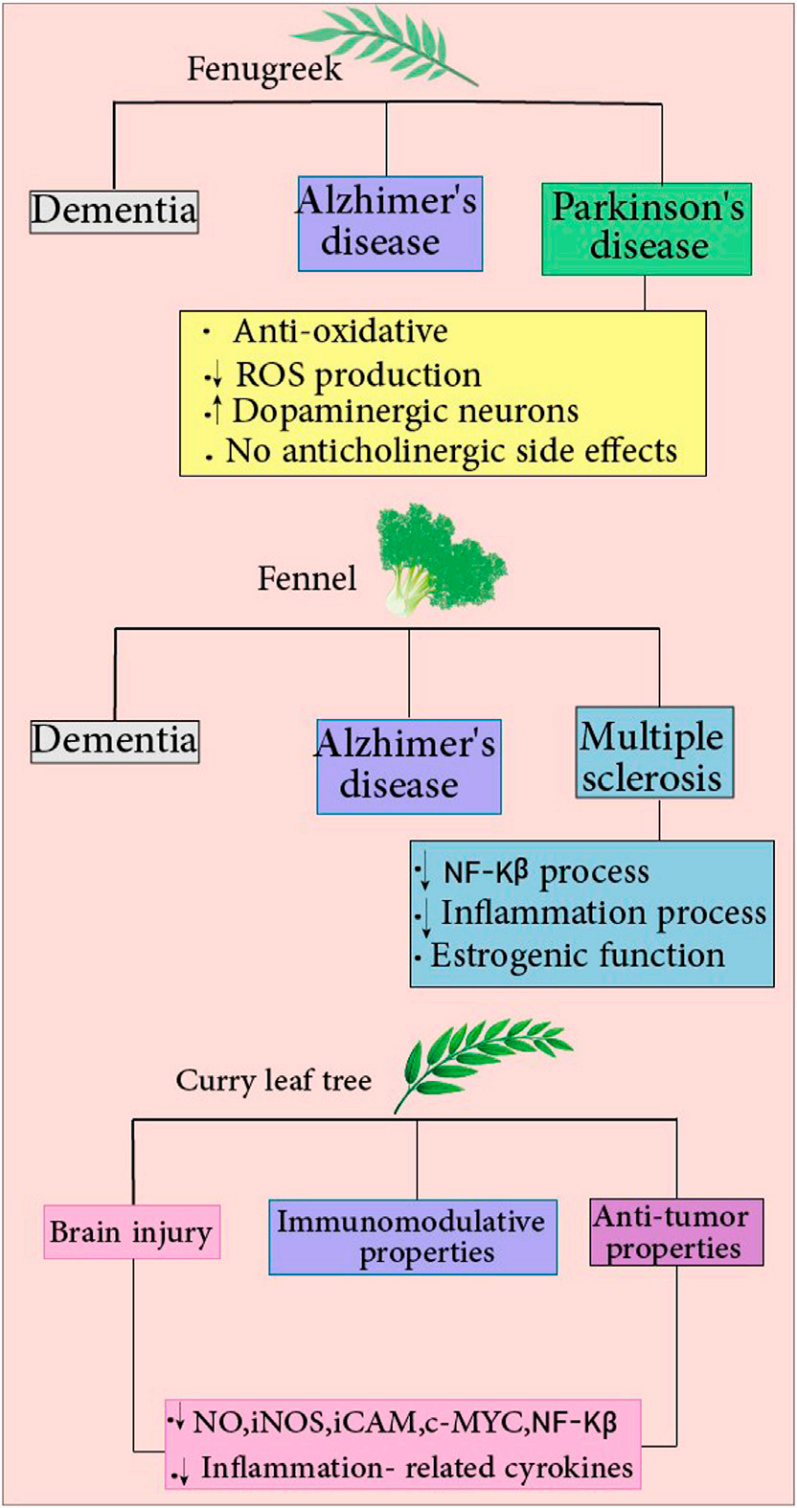
Fenugreek: The compound reduces the risk of various diseases, including Alzheimer’s disease, dementia, and Parkinson’s disease. Fenugreek protects against Parkinson’s disease by reducing ROS production, increasing dopaminergic function in neurons, and providing antioxidant properties with no anticholinergic side effects. Fennel: It reduces the risk of many diseases, including Alzheimer’s, dementia, and multiple sclerosis(MS). Fennel confers protection against MS by decreasing NF-Kβ and inflammation processes and having estrogenic activity. Curry leaf: Different features of this compound include antitumor and immunomodulatory properties. Curry leaf decrease NO, iNOS, iCAM, c-MYC, and NF-Kβ levels and inflammation-related cytokines, which reduce brain injury risks.

*N. sativa* exhibits neuroprotective effects on mice with brain trauma. It was found that total antioxidant status and cell viability were higher in the group that *N. sativa* was administered 1 day before the trauma. Thus, indicating *N. sativa*’s potency in preventing oxidative stress and lipid peroxidation in the presence of brain injury. In addition, oral administration of TQ resulted in reduced lactate dehydrogenase (LDH) activity and MDA levels in neuron nuclei in brains of rats with TBI. Furthermore, the investigators evaluated the anti-apoptosis profile of *N. sativa* histopathologically, using Hematoxylin Eosin (HE) and caspase 3 staining. Apoptotic cells in the brain of mice treated with Nimodipine + *N. sativa* had been less than the group received *N. sativa* alone. However, the authors did not mention the mechanism of the anti-apoptotic effect of *N. sativa*, and only a histopathological essay of apoptotic cell numbers was performed (Mohamed, 2017). TQ (5 mg/kg/day for 7 days) also exhibits neuroprotective and ameliorating effects by improving the neural density in the injured area and decreasing MDA levels in neuron nuclei of TBI rat models (Gülşen et al., 2016). Figure 3. Summarizes the neuroprotective effects of black cumin.

## 5 Black pepper (*Piper nigrum* (L.) [Piperaceae])

Of herbs, black paper is a popular spice, and known for its pungency and bioactive characteristics. It contains different components such as piperine, total phenols, total flavonoids, volatile flavors, and minerals (Lee et al., 2020). Piperin is an anti-inflammatory and antioxidant alkaloid that is extensively used to treat a variety of pathological conditions. Piperin1 (1-piperolpiperidine) is obtained from long pepper (*Piper longum*) and black pepper (*Piper nigrum*) plant seeds. Similar to other alkaloids, piperine is found to have numerous pharmacological effects, such as anti-inflammatory, anti-apoptotic, antioxidant, anti-arthritic, and anti-hypertensive effects (Ravindran and Kallupurackal, 2012).

Black pepper is a known spice and herbal medicine used in many areas. It contains piperine, flavonoids, volatile compounds, and minerals (Lee et al., 2020). Piperin is an anti-inflammatory and antioxidant alkaloid. It is extensively used to treat various pathological conditions. Piperin1 (1-piperolpiperidine) is obtained from long pepper (*Piper longum*) and black pepper (*Piper nigrum*) seeds. Similar to other alkaloids, piperine is found to have numerous pharmacological effects, such as anti-inflammatory, anti-apoptotic, antioxidant, and anti-hypertensive effects (Ravindran and Kallupurackal, 2012). Piperine inhibited pro-inflammatory mediators, IL-6 and PGE_2_ expression and, therefore, has anti-inflammatory effects. Furthermore**,** 100 mg/kg of this substance shows anti-arthritic effects. However, the authors did not investigate the possible mechanism (Bang et al., 2009). Umar et al. reported that Piperine might maintain the cytokine imbalance homeostasis and, therefore, may show protective effects against Rheumatoid Arthritis (Umar et al., 2013). Piprine inhibits several anti-apoptotic mediatrs, including PARP, Bax, caspase-3, caspase-9, and cytochrome c. Thus, it might possess an anti-apoptotic profile (Shrivastava et al., 2013). According to an *in vitro* study, Piperine scavenged hydroxyl radical and was found to be a potent superoxide scavenger with an IC50 of 1.82 mM. The activities mentioned can support the hypothesis that Piperine can be used as an antioxidant substance (Mittal and Gupta, 2000). An *in vivo* study reported that Piperine’s blood pressure lowering effect is known to be related to its possible inhibitory effects on voltage-dependent calcium channels. 1 μM of Piperine inhibited high K^+^-induced contractions in rabbit aorta and therefore is thought to possess Calcium Channel Blockade (CCB) effects (Hlavackova et al., 2010).

### 5.1 Huntington

A study conducted by Salman et al. examined the biochemical and neurobehavioral effects of 3-nitropropioninc(3-NP) acid–induced neurotoxicity and piperine in experimental rats. Piperine (10 mg/kg two times a day for 4 days) treatment improved performance in the treated rats and provided evidence of neuroprotection against 3-NP-produced Huntington-like symptoms. They found that 3NP causes Huntington’s symptoms by impairing motor coordination caused by neural death in the striatum, reducing levels of NT (neurotransmitters), and astrogliosis and increasing the levels of cytokines and inflammatory markers (TNF-α and IL-1β). In another study by Singh et al., (2015), piperine was used as a bioavailability enhancer for curcumin. The 21 days period of treatment with curcumin and piperine resulted in a more significant improvement compared to isolated treatment with curcumin. Additionally, cotreatment with piperine reversed these changes, by enhancing the neurobehavioral performance, rebuilding the level of 5-HT, boosting MAO activity, and decreased neuronal degeneration in striatal tissue in experimental rats. As a result of its modulatory effects, piperine may be useful as a therapeutic agent in the treatment of neuronal damage and dysfunction (Salman et al., 2022).

### 5.2 Parkinson’s disease

Piperine is the primary alkaloid in *Piper nigrum* and has biological attributes, containing intense anti-inflammatory actions. As neuroinflammation has an important effect on Parkinson’s disease the use of piperine might be effective in treating PD. Piperine has been shown to have neuroprotective benefits in PD animal models caused by MPTP or 6-OHDA *via* antioxidant, anti-apoptotic, and anti-inflammatory mechanisms. (Wang et al., 2020). There are several propsed neuroprotective properties for piperine including improved neurodegeneration and memory impairment. Studies have shown that piperine administration in rats improved the function of memory and protected against the degeneration of hippocampal (Mythri et al., 2012; Preethi Pallavi et al., 2018). Correia et al., (2015) examined the neuroprotective effect of piperine(5 and 10 mg/kg for 2 weeks) on PD in an animal model of rats. The results demonstrated that PIP improved the behavioral changes caused by induced PD. It also restored the striatal content of DA and 3,4-Dihydroxyphenylacetic acid (DOPAC), a metabolite of the neurotransmitter dopamine, at higher doses.

### 5.3 Epilepsy

GABA is the major inhibitory neurotransmitter in brain, it has an important role in epilepsy. It is established that Increase of GABAergic neurotransmission is helpful in managing seizure attack (Okada et al., 1989; Rosenstein et al., 1990). Bukhari et al. evaluated the analgesic and anticonvulsant effects of piperine in mice. Piperine (30, 50 also 70 mg/kg) was found to control seizure by mediating opioid and GABAergic pathways (Bukhari et al., 2013). In another study, Fu et al. investigated the effects of piperine on cultured hippocampal neuronal networks. Based on the result of this study, piperine’s neuroprotective effects appear to be related to its role in suppressing synaptic synchronization, Ca2 overload, and presynaptic glutamate release as well as promoting the integrity of intracellular organelles such as mitochondria and Endoplasmic reticulum (Fu et al., 2010). The role of inflammation and oxidative stress in the pathogenesis and exacerbation of disease should not be overlooked. IL-1B is known to be an important inflammatory marker and is found to induce epilepsy by inducing inflammation in the brain (Olgac Dundar et al., 2013). The increased levels of IL-1B could also flare an aggravated inflammatory response including TNF-α release, which itself is able to regulate the synaptic transmission, thus affecting the neural system excitability (Kumar et al., 2007; Tiwari et al., 2012). Initially, oxidative damage was reported to be a consequence of epilepsy, however recent studies suggest that it could play a part in triggering the occurrence of epilepsy (Geronzi et al., 2018). Treatment with piperine as reported by Mao et al., (2017) resulted in reduced duration of seizure and improved memory impairment afterward. This outcome could be explained by the decrease of IL-1B and TNF-α levels, reversing the suppressing effects of Pilocarpine (epilepsy inducer) on antioxidant enzymes including SOD, CAT, and GSH. More importantly, piperine was also found to down-regulate the caspase-3 activity and the expression of Bax/Bcl-2, hence preventing apoptosis and related complications in epilepsy

### 5.4 Stroke

*Pipper nigrum* L. is largely used in treatment of stroke. Hua et al., (2019) studied the impact of dichloromethane fraction (DF) of *Piper nigrum* (100 and 200 mg/kg for 14 days) on rats with induced permanent middle cerebral artery occlusion. Due to induced ischemia, the levels of proteins regulating normal synaptic response including PSD-95 and Syn-l depleted. also the stroke leads to energy metabolism imbalance, fueling a calcium dependent mechanism in which the overexcitement of NMARD receptors with glutamate is followed by an increased influx of Ca2+ and potential neural death (Arundine and Tymianski, 2003; Weilinger et al., 2013). Interestingly, after treatment with DF, there was a dose-dependent rise in the decreased level of PSD-95 and Syn-l. Also the expression of p-CaMK II, CaM, and NR2B proteins increased, which might contribute to prevention of excessive glutamate release, overexcitement of NMARDs and Calcium influx (Rostas et al., 2017).

Regarding the function of these genes, DF improved synaptic plasticity and transmission, which resulted in the attenuation of pMCAO complications such as motor deficiency, neurological dysfunction, postural reflex, body sway, beam balancing, and grip strength. Therefore, DF appears to be beneficial in focal cerebral ischemia treatment. In another study conducted by Zou et al., (2019), the neuroprotective effect of piperine (20 mg/kg, daily for 14 days) was observed in parallel with a decrease in several components of complement and coagulation cascade namely complement component 3, fibrinogen gamma chain, alpha-2-macroglobulin, and serpin family A member 1.

Moreover, in an ischemic state, the mitochondrial imbalance ensues. During the reperfusion phase, mitochondria is involved in producing oxygen free radicals, thus perpetuating the neural damage (BayIr and Kagan, 2008). Also due to ischemia-induced damage, the permeability of mitochondria increase, which disrupts the regulated balance between pro and anti-apoptotic proteins, hence the Cyt c release and caspase cascade activation (Flippo et al., 2018). The alterations in normal mitochondrial activity is sensed by NLRP3 inflammasome complex which leads to cytokine production, mainly IL-1B (Zhou et al., 2011). Interestingly, according to Kaushik et al., (2021) pre-treatment with piperine ameliorated neurological deficits and infarcted area. The underlying mechanism is explained by the role of piperine in restoring the mitochondrial integrity which is followed by inhibition of mentioned complications including ROS production, Cyt c release and apoptosis as well as inflammation. In addition, piperine also exhibited neuroprotective properties *via* regulating apoptosis-related proteins, Bax and Bcl-2 and glial fibrillary acidic protein (GFAP) expression. Also, it preserved the cell viability by restoring the activity of brain derived neurotrophic factor (BDNF) and its transcription protein, cAMP response element binding protein (CREB). Taken together, piperine might be a promising agent to reduce stroke complications in a multimodal manner. In a more recent study, piperine was found to reduce the complications in infarcted area and restore the integrity of hippocampal gyri by inhibiting autophagy through suppressing the PI3K/AKT/mTOR pathway (Zhang et al., 2022).

### 5.5 Brain tumor

Piperine has been studied for its *in vitro* antitumor activity against human cancer cells, however, its specific activity in the case of brain tumors continues to be obscure. Tak et al., (2012) suggested that piperine can be used as a sensitizer to increase the susceptibility of cancer cells to radiotherapy *via* augmenting the ROS production and pro-apoptosis pathways. During radiotherapy cancer cells are exposed to ionizing radiation which induces the production of ROS followed by irreversible damage to vital cellular structures including mitochondria and DNA which eventually lead to apoptosis and cell death (Smets, 1994; Pinthus et al., 2007; Wang and Yi, 2008). Also, the cytotoxicity of piperine was assessed by Sedeky et al., (2018) in which the results indicated that nanomicelles loaded with piperine exert toxicity on human brain tumor cells by inducing apoptosis and cell cycle arrest at G1 phase through reducing the expression of CDK2a, which is necessary for the progression of cell cycle.

### 5.6 Cerebrovascular diseases

Interestingly, several studies have reported promising effects of pepper family derivatives in case of ameliorating risk factors for cerebrovascular diseases and reversing the outcomes such as neural damage. According to Wang et al., (2017) piperine promotes a certain antiatherosclerotic response of body known as Cholesterol efflux (ChE) in macrophages. The buildup of cholesterol in macrophage cells could further lead to formation of atherosclerotic plaques. However, this process can be inhibited by exporting the intracellular cholesterol to other cells (Ohashi et al., 2005; Cuchel and Rader, 2006). In this study, piperine was found to promote ChE by upregulating a prominent cholesterol transporting protein known as ABCA1 (ATP-binding cassette transporter A1) (Phillips, 2014). The underlying mechanism seems to be the inhibitory influence of piperine on calpain activity, a protein which mediates ABCA1 degradation (Wang et al., 2003). One step backward; Dyslipidemia is a result of imbalanced lipid metabolism and increases the risk of atherosclerosis. Thus, Regulating the lipid profile is implied to be a favorable therapeutic target (Rubenfire et al., 2010). Bao et al., (2012) explored the hypolipidemic properties of a novel derivative of piperine named GB-N. The results showed that GB-N improved the lipid profile of hyperlipidemic rats *via* promoting cholesterol transportation from blood to liver. This was attributed the increased activity of LCAT, an enzyme able to esterify free cholesterol for its eventual clearance from blood (Hovingh et al., 2005), and also the upregulated expression of LDLR protein and consequent reduction in LDL plasma level (Go and Mani, 2012). Furthermore, interfering with the blood clotting process has been a practical strategy in order to prevent or treat vascular accidents (Agnoli and Fazio, 1977; Levine et al., 2016). Hence, the potential of Piper nigrum in this area has been the topic of few studies. Piperin (10.75 μl/ml) is reported to have a dose-dependent antiplatelet aggregation *in vitro*, when used in combination with a mixture of other natural compounds present in a traditional indian food RASAM (Devarajan and Raja, 2017). The same outcome was observed when the fruit of *Piper longum* (long pepper) was used in formulation consisting of six other natural products named as Haritaki, Vacha, Rasna, Pippali, Sunthi, Shati, and Pushkaramoola (Unnikrishnan et al., 2015). As mentioned earlier, the properties of pepper family were also investigated in terms of reversing the neural damage and improving related disabilities. Hua et al., (2019) studied the benefits of Dichloromethane Extraction obtained from *Piper nigrum* L. and *Piper longum* L in rats with induced ischemic stroke. This treatment in doses of 100 and 200 mg/kg showed neuroprotective activity in ischemic area *via* preserving the synaptic proteins including syn-I and a-syn and synapses function. besides, since the nerve damage following ischemia can be due to over excitation of neurons and glutamate toxicity (Arundine and Tymianski, 2003), DF treatment in the same dose was found to increase the expression of proteins involved in regulating Ca2+ influx and glutamate release such as PSD-95, p-CaMK II, CaM, and NR2B. Parallel with histological improvement, treated rats had increased body weight and motor ability compared to the control group.

### 5.7 Dementia

Studies investigating the effects of *Piper nigrum* derivatives on dementia are also mainly focused on the ability of these compounds to reverse the cholinergic deficiency, oxidative stress, and inflammatory changes (Chen et al., 2022).

For this matter, Tu et al. (2016) explored the antioxidant and anticholinesterase potentials of five different extracts and 21 alkaloids derived from ethyl acetate extract (EtOAc) *in vitro*. The efficiency of these compounds as anticholinesterases was assessed due to levels of two cholinesterase, Butyrylcholinesterase (BChE) and acetylcholinesterase (AChE) which are found to be involved in promoting cholinergic deficiency and AB aggregation in AD (Talesa, 2001; Darvesh et al., 2003). According to the results, EtOAc was the most potent active extract among others, and out of its derivatives, piperine, piperettine, and piperettyline showed inhibitory action against both AChE and BChE, with piperettine being several times stronger than piperine. This difference might be attributed to the presence of three conjugated double bonds in piperettine. Also, the ability of piperine to inhibit different types of cholinesterase when put together with the evidence from previous studies indicating its role in impairing β-secretase and monoamine oxidase (MAO) function (Rahman and Rahmatullah, 2010; Murata et al., 2015), emphasize its utility in improving memory impairment.

Feruperine, another alkaloid, had the most aggressive activity as a selective BChE inhibitor. Observing the molecular aspect of this action showed that the benzyl ring present in Feruperine forms a strong hydrogen bonding with BChE, explaining the standout inhibitory action of this compound. Moreover, this alkaloid exhibited the highest antioxidant activity and showed superiority in terms of inhibiting AB aggregation than other compounds in this study, including piperine and piperettine. Therefore, constituents of *Piper nigrum*, especially Feruperine can be used to treat AD and dementia as its main symptom through multiple mechanisms.

For better understanding, several studies have focused on the anticholinesterase role of *Piper nigrum* extracts and constituents in animals. Chonpathompikunlert et al. (2010) administered different doses of piperine to rats with chemically induced AD. Piperine has been found to be effective in several ways including slowing down the neurodegeneration process, improving memory function, and inhibiting AChE activity. A 3-month-long treatment with daily doses of methanolic extract of *Piper nigrum* was found to have similar results in another study (Ahmed et al., 2013). It is worth noting that the results of the first study indicated possible neurotrophic activity for this compound, since piperine, especially at lower doses, improved the density of neurons located in the hippocampus (Chonpathompikunlert et al., 2010). However, further studies are warranted to draw a conclusion on the neurotrophic properties of piperine.

As mentioned earlier, damping the inflammation process is one of the main targets for anti-AD drugs. Concerning this matter, Ahmed et al. (2013) measured the levels of some pro-inflammatory molecules in rats with induced AD, before and after treatment with methanolic extract of *Piper nigrum*. The Rats were given daily doses of *Piper nigrum* extract (187.5 and 93.75 mg/kg) for 3 months. the methanolic extract exhibited significant ability to reduce the increased levels of inflammatory mediators, being C-reactive protein (CRP), total nuclear factor kappa-B (NF-κB), and monocyte chemoattractant protein-1 (MCP-1).

The benefits of methanolic extract of *Piper nigrum* are not narrowed to only anti-inflammatory and anticholinesterase activity. Two separate *in vivo* studies suggested that this extract also acts as an antioxidative agent *via* interfering with the superoxide dismutase and Catalase function as well as promoting Glutathione peroxidase activity, leading to decreased lipid peroxidation and ROS mediated damage in the hippocampus of Rat models of AD. Also, Vurmaz et al. (2020) reported a significant decrease in levels of Malondialdehyde, a biomarker for oxidative stress, in the liver and plasma of AD-induced rats after receiving piperine (10 mg/kg/day for 5 weeks). The results of this study, further included the effectiveness of piperine in terms of reducing the degenerative area and mRNA expression of MAPK-1 gene in hippocampus. This gene encodes the homonymous enzyme which promotes cell death and has found to be elevated in AD brains. the positive outcomes of this treatment were further supported by the improved memory performance of the treated group in behavioral tasks (Hritcu et al., 2014; Hritcu et al., 2015).

Similar to *Piper nigrum*, there are also other natural compounds explored for their possible anti-dementia activity, therefore some studies investigated the concomitant use of *Piper nigrum* derivatives and other herbal medicines. In the study of Teymuori et al. (2021), *Piper nigrum* was administered at doses of 50 and 100 mg/kg alone and together with Cinnamon zeylanicum (CZ). Similar to *Piper nigrum*, CZ is also proposed to be a promising natural product with anti-AD features (Frydman-Marom et al., 2011; Jain et al., 2015). The memory performance of rats with induced AD was further analyzed by two different memory tests, the passive avoidance test (PAT) and the object recognition test (ORT). In the PAT, rats showed slight improvement only when compounds were used in the highest available doses in this study, (*Piper nigrum*: 100 mg/kg, CZ:400 mg/kg), unaffected by the combined use. However, the recognition index in the ORT model significantly increased in rats that have received *Piper nigrum* (50 mg/kg) and CZ (400 mg/kg) simultaneously compared to those who only received monotherapy. similarly, Gupta et al. (2013) reported that the addition of *Piper nigrum* powder to Bacopa monnieri or Withania somnifera, two herbs exhibiting memory enhacing activity (Pase et al., 2012; Choudhary et al., 2017), significantly improved the effects of these substances to decrease and reverse neural loss induced by AChE activity also, it is found that the concurrent ingestion of piperine (20 mg/kg-daily for 28 days) and curcumin (100 and 200 mg/kg), a natural antioxidant, exhibits protection against cognitive decline and oxidative stress in mice (Rinwa and Kumar, 2012). In addition the sole effects of each compound, the augmented anti-dementia activity could be explained by the increased absorption rate and bioavailability of curcumin as *Piper nigrum* affects curcumin metabolism (Shoba et al., 1998).

### 5.8 Alzheimer’s disease

In general, phytochemicals are found to reverse the AB aggregation and Tau hyperphosphorylation through multiple mechanisms. Phytochemicals are reported to be inhibitors of the amyloidogenic monomers production as well as oligomeric and fibrillar aggregates, with inducing proteolytic system activity, the neuronal function could also be restored (Smid et al., 2012; Bieschke, 2013). Also, based on the pathological process, inhibiting the activity of β-secretase (BACE1), an enzyme promoting the breakdown of APP and AB plaque formation, could be an effective therapeutic target (Yan and Vassar, 2014). Interestingly, many phytochemicals are found to inhibit this enzyme, including Curcumin which its bioavailability and effectiveness is increased in case of co-treatment with piperine (Shoba et al., 1998; Thapa et al., 2016; Naoi et al., 2019). It is worth noting that Curcumin and other phytochemicals has also shown the ability to inhibit Tau hyperphosphorylation (Park et al., 2008; Chua et al., 2017).

Phytochemicals influence the expression of neurotrophic factors (NTFs), such as brain-derived and glial cell line-derived NTF (BDNF, GDNF), which are crucial for the viability and function of neurons and if reduced, could promote the development of AD (Siegel and Chauhan, 2000).

Piperine was found to enhance BDNF in animal models of aging, depression, Alzheimer’s disease, PD, stroke, brain ischemia and showed neuroprotection (Naoi et al., 2019). In a study by Chonpathompikunlert et al. (Chonpathompikunlert et al., 2010) piperine at different doses (five also 10 and 20 mg/kg for 2 weeks) was given to animal models both prior to and after inducing AD. interestingly, piperine showed to have positive effects on memory impairment and hippocampal neurodegeneration. This could possibly be assigned to piperin’s neurotrophic properties and its role in reducing lipid peroxidation and AchE enzyme activity. Similarly, Wang et al. (2019) reported decreased cognitive decline due to antioxidant activity for piperine (2.5–10 mg/kg/daily/for 15 days) was along with its role in ameliorating neuroinflammation and impaired neurotransmission in the hippocampus caused by AD. The main mechanism of neurodegeneration is neuroinflammation. The main feature of neuroinflammation is the activation of microglia, which releases neurotoxic substances and pro-inflammatory cytokines, resulting in the death of neurons. In order to alleviate the neuroinflammatory processes associated with neurodegeneration, suppression of the overactivation of microglia with novel pharmacological agents is a promising approach (An et al., 2020). On the subject of anti-inflammation potential of piperin, Roshanbakhsh et al. (2020) observed the depletion of pro-inflammatory mediators such as TNF-α, IL-1β, and NF-κB signaling pathway, iNOS expression, and glial activation in the hippocampus of AD animal models treated with piperin (5, 10, 20 mg/kg–every day for 10 days). This was followed by the restraint of demyelination process, repair of myelin damage, and better memory performance. The result of this study was in line with Piperine attenuating the inflammatory response induced by LPS *via* inhibiting NF-κB activation and enhancing the Nrf2 signaling pathway in BV2 microglia, in another study (An et al., 2020). Moreover, since NMDA (N-methyl-D-aspartate) receptor antagonists are a family of medications that may be effective in the treatment of AD, piperine (25–86) mg/kg) can help treat AD due to its NMDAR antagonist action (D'Hooge et al., 1996).

Methanolic extract of Piper nigrum is found to possess remarkable antioxidant properties. According to Hritcu et al. (2014) treatment with the methanolic extract improved memory performance in rat models of AD through an antioxidant process in order to limit the oxidative damage caused by amyloid beta accumulation. This process involves the rise in the levels of decreased antioxidant enzymes and reducing the intracellular formation of H2O2 as well as lipid peroxidation (10 ml/kg, evevry day, for 21 days). It is reported that *Piper nigrum* extract also exhibit anti-inflammation properties. According to (Ahmed et al., (2013) AD-related increased brain AchE levels and pro-inflammatory mediators such as C-reactive protein (CRP), total nuclear factor kappa-B (NF-κB), and monocyte chemoattractant protein-1 (MCP-1), significantly decreased after treatment with Piper nigrum extract (187.5 also 93.75 mg/kg. every day for 3 months).

Limonene is a monoterpene that belongs to the Rutaceae family and a rich natural compound in antioxidant, anti-inflammatory properties. Several investigations have shown that limonene has neuroprotective properties in neurodegenerative disorders. Interestingly, limonene is also a constituent of Piper nigrum (Eddin et al., 2021). The natural bicyclic sesquiterpene b-caryophyllene (BCP) is a significant volatile plant component found in high proportions in the essential oils of several species, including Piper nigrum. BCP has been shown to have anti-inflammatory, antiviral, and antioxidant properties in recent research (Prabhakar and Govil, 2015). BCP preferentially binds to cannabinoid receptor type 2 (CB2R) and functions as a complete agonist, according to the research. Interestingly, the cannabinoid system is involved in neuroinflammation and neurodegeneration (Assis et al., 2014).

### 5.9 Memory

Saeri et al. (2020) evaluated the effects of the regiment consisting of *Piper nigrum*, *Cyperus rotundus*, *Crocus sativus*, and *Boswellia serrata* on learning and memory deficit caused by hypothyroidism in rats. This regiment was given at two doses (640 and 1,280 mg/kg) for 6 months. The comparison between two groups of case and control showed that the syrup of the herbal plants preserve brain and neural tissue from the oxidative damages, attenuates memory impairment and tissue damage induced by hypothyroidism. Figure 3. Summarizes the neuroprotective effects of black pepper.

## 6 Curry leaf tree (*Murraya koenigii* (L.) Spreng [Rutaceae])

M*urraya koenigii* Spreng or curry leaf, a member of the Rutaceae family, is a popular Asian herb, which is usually used to enhance the flavor of curries and fried foods (Mohd Nor et al., 2009; Rao et al., 2011). Notably, *M. koenigii* contains a bunch of bioactive compounds with promising antioxidants properties such as mahanine, mahanimbine, isolongifolene, koenimbine, girinimbine, isomahanine, koenoline, and O-methylmurrayamine (Gil and Sharma, 2014; Rehana et al., 2017). In fact, antioxidant activity of *M. koenijii* may result from hydrogen or electron transfer, metal chelating, and synergistic activity (Gupta and Sharma, 2010). In recent years, identification and development of phenolic compounds have been attracted great attention relying on their strong antioxidant capacity (Ramkissoon et al., 2013). Seemingly, phenolic compounds, especially phenolic acids and flavonoids, are major agents responsible for antioxidant and pharmacological properties of such plants (Ignat et al., 2011; Zhang et al., 2011).

### 6.1 Brain injury

It attenuated the levels of nitric oxide, inflammation-related cytokines, and other gene products including iNOS, iCAM, NF-kB, and c-MYC. Regarding the similar pathogenic base for various types of cancers, consumption of *M. koenigii* might be a savior alternative in the field of chemotherapeutics for brain tumors, too. Therefore, further research on this topic is warranted.

Also, proteins involved in the immune system play critical roles in the normal development of the brain, and reasonably, malfunctioning of such proteins may result in neurological diseases (Kuehn, 2008).

### 6.2 Antitumor properties

Furthermore, it attenuated the levels of nitric oxide, inflammation-related cytokines, and other gene products including iNOS, iCAM, NF-kB, and c-MYC. Regarding the similar pathogenic base for various types of cancers, consumption of *M. koenigii* might be a savior alternative in the field of chemotherapeutics for brain tumors, too. Therefore, further research on this topic is warranted.

### 6.3 Immunomodulative properties

**FIGURE 4:**
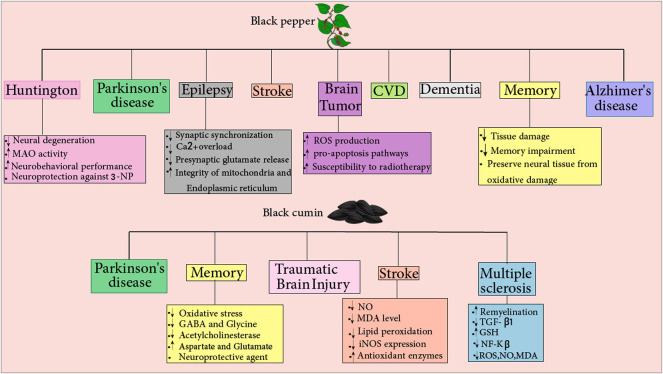
Black pepper: this compound reduces the risk of various diseases, such as Huntington’s, Parkinson’s, epilepsy, stroke, brain tumor, CVD, dementia, memory loss, and Alzheimer’s disease. Black pepper protects against Huntington’s by reducing neural degeneration and MAO activity and increasing neurobehavioral performance. Additionally, black pepper leads to neuroprotection against 3-NP. Black pepper prevents epilepsy by reducing synaptic synchronization, Ca2+ overload, and presynaptic glutamate release and increasing integrity of mitochondria and endoplasmic reticulum. It also prevents brain tumor by increasing ROS production and pro-apoptosis pathways and increasing susceptibility to radiotherapy.By reducing tissue damage, memory impairment, and preserving neural tissue from oxidative damage black pepper protects against memory loss. Black cumin: This compound reduces the risk of various diseases, such as Parkinson’s, memory loss, traumatic brain injury, stroke, and multiple sclerosis.It prevents memory loss by reducing oxidative stress, GABA, glycine, and acetylcholinesterase and increasing aspartate and glutamate. Also, black cumin is a neuroprotective agent. Black cumin prevents stroke by reducing NO and MDA level, iNOS expression, and lipid peroxidation and increasing antioxidant enzymes. The black cumin protects against multiple sclerosis by increasing remyelination and GSH and reducing NF-Kβ, ROS, NO, MDA, and TGF-β1.

Also, proteins involved in the immune system play critical roles in the normal development of the brain, and reasonably, malfunctioning of such proteins may result in neurological diseases (Kuehn, 2008). Figure 4 summarizes the neuroprotective effects of curry leaf.

## 7 Fenugreek (*Trigonella foenum* graecum (L.) [Fabaceae])

Fenugreek [*Trigonella foenum* graecum (L.) (Fabaceae)] is a leafy plant and flavor that originated in Eastern Europe; however, it is currently cultivated worldwide (Max, 1992). Some pre-clinical and clinical investigations have shown that it can treat a number of pathological disorders, thus gaining the attention of many researchers. There are several chemical ingredients in fenugreek grains that contribute to its nutritional value and therapeutic properties. These ingredients include dietary fiber, proteins and vitamins, flavonoids, alkaloids, coumarins, vitamins, and saponins, which have turned fenugreek to one of the well-known herbal medicines (Jayaweera, 1981).

### 7.1 Dementia and Alzheimer’s disease

Several studies explored the potential role of fenugreek in reversing or decelerating AD progress. It is commonly observed that Fenugreek seed extract (FSE) is able to inhibit the activity of acetylcholinesterase (AChE), a prominent element in the pathogenesis of AD. Prema et al. (2017) reported that this AChE inhibitory activity is associated with FSE-mediated activation of the Akt/GSK3β pathway, which also led to ameliorated memory and learning difficulties, Al toxicity, AChE hyperactivity, amyloid β (Aβ) aggregation, and apoptosis, in rats with AD-like AlCl3–induced memory loss in this study. Interestingly, FSE was also found to have therapeutic effects on rats with AlCl3 toxicity (mimicking AD) through an anti-inflammatory and anti-oxidative process. Which enhanced the activity of antioxidant enzymes (GSH, CAT, SOD), reduced the levels of inflammatory mediators such as Iba-1, IL-1β, IL-6, TNF-α, iNOS, NF-κB, COX-2, and CDK5-mediating tau pathology. FSE also increased the immune content of BDNF and STAT3, promoters of cell survival, which may assign to its antioxidative properties.

Also, a growing number of studies proposed that hypercholesterolemia is a causative risk factor that sensitizes patients to pathologies such as AD, since high levels of cholesterol promotes Aβ accumulation, hyperphosphorylation of tau (pTau), and cognitive dysfunction (Sparks et al., 1990; Popp et al., 2013). As previously mentioned, it has been shown that fenugreek seeds (10 also 20% fenugreek seeds for 9 weeks), contain soluble dietary fibers, which by decreasing total cholesterol, low-density lipoprotein cholesterol (LDL-C), and triglycerides in the blood and liver provide antihyperlipidemic actions (Ramulu et al., 2011).

### 7.2 Parkinson’s disease

Individuals experiencing PD may benefit from the supplementary treatment of fenugreek combined with L-Dopa. According to Nathan et al. (2014) fenugreek (300 mg, two times a day for 6 months) as an adjuvant to L-Dopa improved the scores of Unified PD Rating Scale (UPDRS), and Hoehn and Yahr (H&Y) staging compared to control group which received the regiment of placebo and L-Dopa. Favorably, fenugreek was also found to have superior safety and tolerability profile, making the supplementation more efficient. In another study by Gaur et al. (2013) the reversal of motor symptoms were observed when using a standardized hydroalcoholic extract of fenugreek (10, 30 or 100 mg/kg). The authors suggested that this effect can be assigned to fenugreek’s anti-oxidative and ROS scavenging activities as well as its role in enhancing dopaminergic neurons. It is worth noting that this extract of fenugreek displayed no anticholinergic side effects which magnifies the previous benefits since the anticholinergic activity of the conventional drugs for PD is considered a major problem. Figure 4 summarizes the neuroprotective effects of fenugreek.

## 8 Fennel (*Foeniculum vulgare* Mill [Apiaceae])

Fennel (*Foeniculum vulgare* Mill [Apiaceae]), belongs to the family Apiaceae (Umbelliferaceae), and is a native plant to central Europe and Mediterranean region. However, it is cultivated throughout the world, since its aromatic fruits can be used for food seasoning (Díaz-Maroto et al., 2006). as a medicinal herb, fennel has been explored by multiple studies (mentioned below) to determine the neuroprotective potential this herb in different preparations, with methanolic extracts and essential oils being the most common. The chemical aspect of fennel’s essential oil typically consists of trans-anethole, fenchone, estragol (methyl chavicol), and α-phellandrene (Díaz-Maroto et al., 2006).

### 8.1 Alzheimer’s Disease and Dementia

The neuroprotective effect of Fennel (*Foeniculum vulgare* Mill.) on AD has been investigated by several studies. An *in vitro* study by Mizuno et al. (2015) was performed to determine the role of essential oils (centration was 25 ppm and for 24 h) including Fennel on neuronal death elicited by aluminum, zinc, hydrogen peroxide (H_2_O_2_), and the estrogen receptor antagonist (tamoxifen). Essential oil of fennel was found to attenuate the neuronal death induced by H_2_O_2_. Fennel remarkably increased the viability of hypothalamic neuronal cells (GT1–7 cells) exposed to H_2_O_2_ and showed neuroprotective properties against toxicity in a dose-dependent manner.

Another study by Joshi and Parle (2006) was performed to determine the anticholinesterase and neurotrophic potentials of Foeniculum vulgare Mill. Linn (50 also 100 and 200 mg/kg, for 8 days) in mice with scopolamine induced memory loss. eight consecutive days of administering Methanolic extract of F. Vulgare Linn. ameliorated the scopolamine amnesic effect (0.4 mg/kg) and memory deficits. To assess the memory, the passive avoidance paradigm was used as an exteroceptive behavioral model. Observations showed that F. Vulgare extract significantly increased acetylcholinesterase inhibition and step-down latency in mice. This may indicate the underlying mechanism for improved memory performance. As several other studies also reported the same alterations followed by Fennel treatment. for instance, Bhadra et al. (2011) reported similar Acetylcholinesterase (AChE) and Butyrylcholinesterase (BChE) inhibitory activity for fennel essential oil (0.5–25 μg/ml). Moreover, halting the oxidative stress process appears to be a recurring finding in studies exploring the features of fennel preparations. Bhatti et al. (2018), investigated possible neuroprotective effects of fennel extract ethanol in a mice model of lead-induced neurotoxicity. It was observed that depleted levels of oxidative stress markers (SOD1 and peroxiredoxin-6) and the three isoforms of APP (APP common, 770 and 695) expression nearly reached the normal levels in the hippocampus and cortex of the fennel extract-treated mice, especially at the dose of 200 mg/kg/day for 21 days. also, histological findings showed that morphological abnormalities were remarkably ameliorated after treatment with fennel extracts. A study by Koppula and Kumar (2013) found that the level of urinary vanillylmandelic acid induced by stress, remarkably reduced by administering fennel extract (50, 100, and 200 mg/kg/day) in rats. Fennel also improved memory deficits induced by scopolamine in rats in a dose-dependently manner. The fennel extract also showed potential inhibitory effects on lipid peroxidation in both the brain and liver of rats when compared to ascorbic acid as a standard antioxidant.

### 8.2 Multiple sclerosis

Anethol, a compound found in fennel, is believed to have estrogenic function, hence suppressing the inflammation process (Albert-Puleo, 1980). This effect may be *via* the inhibition of inhibitory subunit of NF-kB (IκBα) degradation which leads to the suppression of the nuclear transcription factor KB (NF-κB) pathway (Sen et al., 1996; Chainy et al., 2000). Studies have showed that NF-κB plays a role in mediating multiple sclerosis and other inflammatory diseases (Huang et al., 2002). Thus, consuming fennel in these patients might have beneficial effects.

Figure 4 summarizes the neuroprotective effects of fennel.

## 9 Coriander (*Coriandrum sativum* (L.) [Apiaceae])

Coriander is a natural spice isolated from a plant belonging to the Umbelliferae (Apiaceae) family. For centuries, Coriander and its extracts has been considered a remedial herb helping with various ailments including gastrointestinal and respiratory diseases, pain, infections, and memory (Burdock and Carabin, 2009; Sahib et al., 2013). The essential oil of coriander is mostly used for therapeutic aims due to being composed of linalool (majorly), alcohols, ketones, and esters such as α-pinene, γ-terpinene, geranyl acetate, and camphor (Nejad Ebrahimi et al., 2010). These compounds have been found to ameliorate several neurological conditions, which are described below.

### 9.1 Alzheimer’s disease

The neuroprotective effect of coriander on AD has been investigated by several studies. In a study by Caputo et al. (2021), the neuroprotective potential of coriander and its main active portion linalool was investigated against the neurotoxicity of β-amyloid protein (Aβ) 1–42 oligomers, the primary molecular trigger in the neurodegeneration of AD. Their findings indicate that coriander essential oils (EOs) and linalool at a concentration of 10 μg/ml have the capability of improving viability and reducing the nuclear morphological abnormalities of cells treated by Aβ1–42 oligomers for 24 h. Additionally, coriander EOs and linalool have been shown to counteract the increased intracellular production of reactive oxygen species and the activation of an enzyme induced by Aβ1–42 oligomers named the pro-apoptotic caspase-3 enzyme. It is important to mention that their results on distinct PC12 neurons exposed to Aβ1–42 oligomers are consistent with the results of two other studies describing similar neuroprotective properties for coriander essential oils (EOs) and linalool. Therefore, coriander EOs and its main portion, linalool, could act as a natural agent of remedial interest against the neurotoxicity induced by Aβ1–42. In addition, it is documented that the coriander extract is able to inhibit the receptor of epidermal growth factor and the extracellular signal-regulated kinase (ERK) phosphorylation induced by Aβ. The active form of ERK was found to abolish the protective effect of coriander extract against toxicity induced by Aβ42, hence indicating the existing link (Liu et al., 2016).

Cioanca et al. (2013) Suggested that coriander’s memory-enhancing effect and neuroprotection against Aβ-induced injuries are through maintaining a balance between antioxidant enzymes, SOD and GSH. following the inhaling of, SOD activity reduced in parallel with the increase of GSH activity. This resulted in inhibiting lipid peroxidation and decreasing the hippocampal level of its products such as MDA. In this study, volatile oil form of *C. sativum* was also found to reduce LDH activity, which is known to be an indicator of tissue damage (Drent et al., 1996). Interestingly, inhaled volatile oil form of C. sativum L. (200 μL/60 min exposure, every day, for 21 days) was also shown to maintain the similar balance between CAT and GSH (desrease and increase, respectively), resulting in the better performance in assessment tests such as plus-maze test and forced swimming test (Cioanca et al., 2014). moreover, *Coriandrum sativum* leaves (CSL) (five also 10 and 15% w/w for 45 days) has shown dose-dependent effectiveness in case of lowering serum total and exerting anticholinesterase activity and improving memory impairment (Mani et al., 2011).

### 9.2 Epilepsy

Epilepsy is a central nervous system condition with higher new cases rate in children and elderlies. Individuals with epilepsy often suffer from physical problems such as bony fractures and tend to have higher rates of mental disorders. Epilepsy is characterized by experiencing recurrent unprovoked seizures. Symptoms might vary depending on the area of the brain that is involved. Loss of consciousness, impairment in cognitive function and movement disturbance are among common symptoms. Abnormal activity of cortical neurons is responsible for initiating seizure and axons and glial cells might be involved. Epileptogenic networks are distributed widely in generalized epilepsy and can involve thalamocortical structures. In focal epilepsy, disruption in neural networks is usually in one hemisphere (Thijs et al., 2019).

Epilepsy is among the most common neurological conditions in older people. Its intermittent and unpredictable nature make epilepsy more important for this group of people. Physical injury is more frequent in elderlies as a result of the increased tendency for falls. Neurodegenerative conditions, head injuries and brain tumors are the most known causes of epilepsy in older patients with epilepsy (Johnston and Smith, 2010).

Cilantro (*Coriandrum sativum* L.) has been demonstrated to have antiepileptic activities. Investigators found that (E)-2-dodecenal, a metabolite found in cilantro oil, is a potent neuronal voltage-gated potassium channel subfamily Q (KCNQ) activator. Dysfunction of this subfamily has been previously identified as a cause of epileptic encephalopathies. (E)-2-dodecenal promotes KCNQ2/3 opening at 100 nM and can delay seizures induced by pentylene tetrazole (Manville and Abbott, 2019).

## 10 Cumin (*Cuminum cyminum* (L.) [Apiaceae])

Cumin (*Cuminum cyminum* (L.) [Apiaceae]) is an indigenous plant of Mediterranean region, belonging to the Umbelliferae (Apiaceae) family (Hajlaoui et al., 2010). Cumin seeds are mostly known as a popular food additive with strong aroma and cultivated in various regions (Zohary et al., 1995; Gohari and Saeidnia, 2011). In addition its use in food seasoning, cumin has been present in the list of traditional medicines for ages. Cumin’s therapeutic potential for toothaches, epilepsy, and gastrointestinal maladies has been discussed the most (Mnif and Aifa, 2015). however, it seems cumin and its extracts may also be favorable choices for neurological disorders.

### 10.1 Alzheimer’s disease

The neuroprotective effect of Cumin (*Cuminum cyminum*) on AD has been investigated by several studies. Kumar and Chowdhury (2014) reported an Acetylcholinesterase (AChE) inhibitory activity for aqueous extract of C. cyminum fruit at dose of 50 μg/ml. According to Koppula and Choi (2011), it was found that oral administration of cumin aqueous extract (at doses of 100, 200, and 300 mg/kg/day) remarkably attenuated amnesia induced by scopolamine in rats. enhanced memory activity was characterized by improved retention, acquisition, and recovery in rats treated with extract in comparison with the control group. The extract also remarkably inhibited lipid peroxidation compared to ascorbic acid (a well-known antioxidant) in both the liver and brain of rats.

### 10.2 Dementia

Dementia is a clinical syndrome and a progressive devastating disease characterized by decline in cognition, behavioral changes function disability. Cerebral cortex injuries are responsible for impaired cognition. Other symptom are memory loss, agnosia, communicating difficulty, and language impairment. Agitation, psychosis and apathy are among common behavioral changes in dementia. (Duong et al., 2017). It is estimated that rate of dementia doubles from 65–90 years showing that dementia risk is associated with age and aging acts as a proxy for pathophysiological processes that are factors for the dementia onset. (Rosa et al., 2020).

Cumin fruit extract has been reported to be an antioxidant and AChE inhibitor. It has been reported to have potential effects on enhancing memory, improving recovery and reducing scopolamine-induced amnesia in rats. Furthermore, cumin extract can inhibit lipid peroxidation in rat brain and liver. Thus it is suggested that cumin might have beneficial effects on dementia. (Iranshahy and Javadi, 2019).

### 10.3 Parkinson’s disease

Morshedi et al. (2015) conducted a study to investigate the Cumin essential oils inhibitory effects on the fibrillation of α-SN. α-synuclein depositions are present in the brain of PD patients, forming the well-known lewy bodies. Cuminaldehyde fraction of the Cuminum cyminum L. cyminum was observed to interfere with proteins assembly into β-structural fibrils, which might be due to the interaction of cuminaldehyde’s aldehyde group with amine groups. favorably, no toxicity related to the fraction was found in PC12 cells. In addition, Kim et al. (2016) reported that oral administration of cumin aqueous extract (at doses of 100, 200, and 300 mg/kg/day) remarkably improved MPTP-induced cognitive and locomotor deficits in mice when prescribed for 3 weeks. Cumin also remarkably elevated the decreased levels of antioxidant enzyme (superoxide dismutase (SOD) and catalase) and also inhibited lipid peroxidation induced by MPTP in brain tissues of mice.

### 10.4 Epilepsy

Essential oil extracted from *Cuminum cyminum* L. (Doses of 0.05 and 0.15 and 0.25 and 0.50 ml/kg) after has been demonstrated to have anticonvulsant and sedation activity. Sayyah et al. reported that an essential oil obtained from *Cuminum cyminum* mediates sedation and motor relaxation in mice with seizures induced by pentylene tetrazole or maximal electroshock, a dose-dependent manner (Sayyah et al., 2002).

## 11 Cardamomum (*Elettaria cardamomum* (L.) Maton [Zingiberaceae])

Cardamomum (*Elettaria cardamomum* (L.) Maton [Zingiberaceae]) as a fruit of *Elettaria cardamomum*, naturally growing plant in south Asia and belonging to the Zingiberaceae family (Govindarajan et al., 1982). For ages, cardamom has been used to treat several ailments, most notably gastrointestinal diseases (Hamzaa and Osman, 2012; Saeed et al., 2014). The therapeutic potential of this herb is ascribed to the essential oil and its bioactive components. The essential oil of cardamom contains abundantly monoterpene components, such as 1,8-cineole, α-pinene, α-terpineol, linalool, linalyl acetate, nerolidol and α-terpinyl acetat (Yashin et al., 2017; Ashokkumar et al., 2020).

### 11.1 Alzheimer’s disease

**FIGURE 2:**
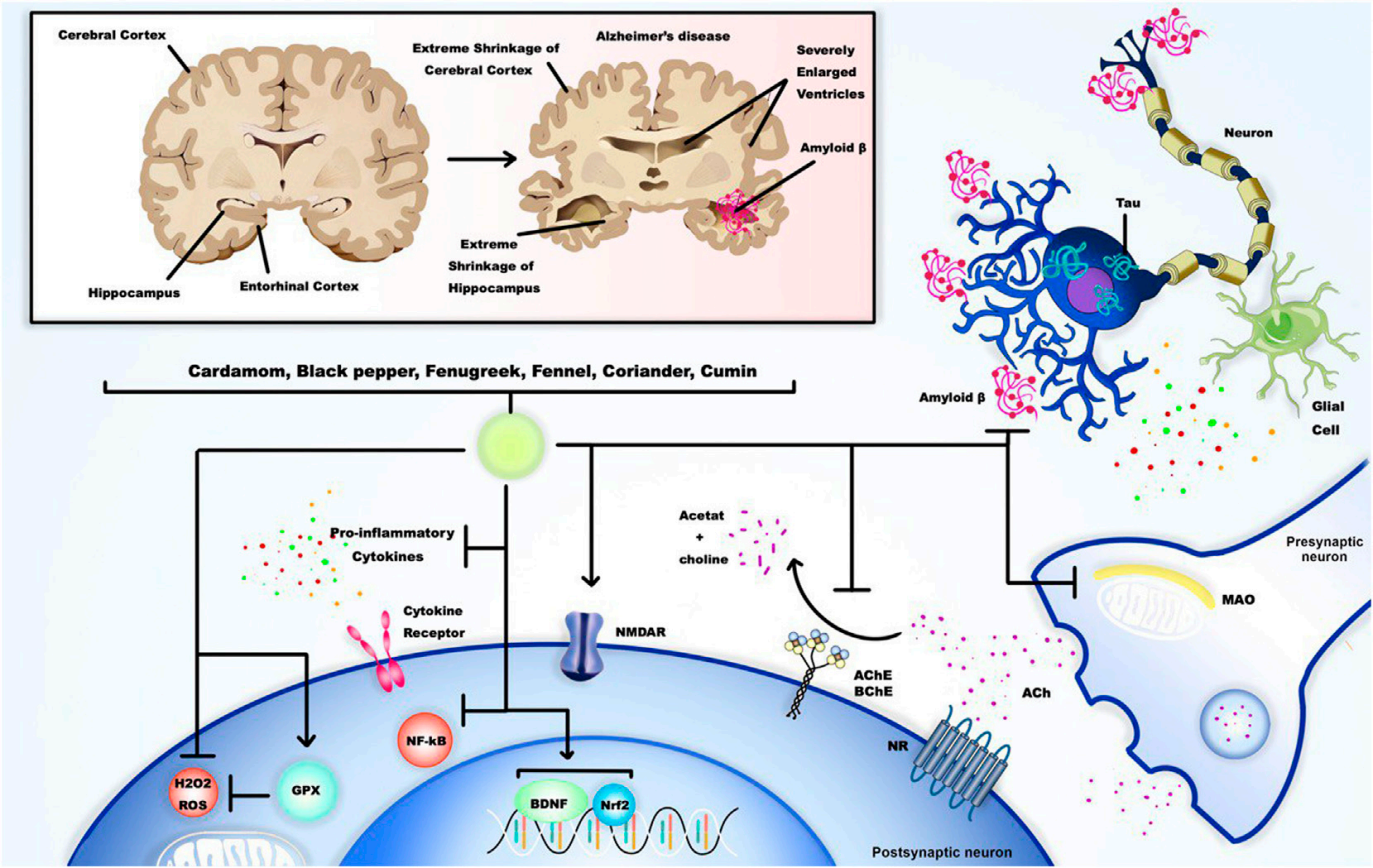
Alzheimer’s disease (AD), as a neurodegenerative disease, is the most common form of dementia. Common Indian spices such as cardamom, black pepper, fenugreek, fennel, coriander, and cumin all have neuroprotective effects in AD through inhibition of amyloid (Aβ) plaques formation, inflammation, and NF-κB pathway, MAO formation, H2O2 and ROS formation, and butyrylcholinesterase (BChE) and acetylcholinesterase (AChE) activity. These components can also induce NMDA (N-methyl-D-aspartate) receptor function, glutathione peroxidase (GPX) activity, and Nrf2 and BDNF transcription.

The anti-cholinesterase and antioxidant activity of cardamom (*Elettaria cardamomum*) extract) (500 and 1,000 mg/kg) for 15 days (as well as significant induction of endogenous antioxidants such as glutathione and superoxide dismutase) may explain its beneficial effects (Kunwar et al., 2015). In one study, Gomaa et al., (2019) evaluated the effects of cardamom (25 mg/kg) on AD-induced alterations in an animal model. They concluded that such therapy could alleviate AD-like changes through stimulation of attenuated insulin signal transmission in the brain, mitigation of related oxidative stress, and neuroinflammation. According to Paul et al., 1,8-cineole-rich extract (50 and 100 µM) of small cardamom seeds interfered with the AD-related pathological events *via* impeding the production of reactive hydroxyl radicals, preventing the formation of Aβ42 deposits, and protecting cells from iron-induced death (Paul et al., 2020). Figure 2 summarizes the effect of common Indian spices on neurological disorders.

## 12 Conclusion and future prospective

This review intends to highlight the links between common Indian spices and treatments of age-related neurological disorders. Based on the data available, the association between eight Indian spices (black cumin, black pepper, curry leaf tree, fenugreek, fennel, coriander, cumin, and cardamom) and several age-related neurological diseases such as Parkinson’s disease, Alzheimer’s disease, dementia, epilepsy, multiple sclerosis, cerebrovascular disorders, traumatic brain injury, Huntington’s disease, and brain tumor was summarized. The proposed mechanisms are presented in order of spice investigated in this review:

Neuroprotective effects of black cumin (Nigella sativa) are shown in several age-related neurological diseases including Parkinson’s disease, memory loss, multiple sclerosis, stroke, and traumatic brain injury. The following mechanisms have been proposed for the potential neuroprotective effects of this spice: decrement in the expression of synaptophysin and the synaptic vesicle recycling increment; upregulating the Nrf2/ARB signaling pathway; subsequent increase in antioxidant enzymes expression such as glutathione-S-transferase (GST), quinone oxidoreductase (NQO1), and heme oxygenase 1 (HO-1); reducing the LDH release; inhibiting acetylcholinesterase (AChE); increasing 5-HT levels; preventing ROS production by decreasing NO and MDA; suppressing TGF β1 expression; inhibiting NF-ĸB activation; inhibiting lipid peroxidation; elevating reduced activity of antioxidant enzymes (SOD, GSH, and CAT); and upregulating vascular endothelial growth factor (VEGF) gene expression, matrix metallopeptidase (MMP9), and hypoxia-inducible factor-1 (HIF).

Black pepper is shown to have neuroprotective effects against several age-related neurological diseases including Huntington, Parkinson’s disease, epilepsy, stroke, brain Ttmor, cerebrovascular diseases, dementia, Alzheimer’s disease, and memory loss. The proposed mechanisms are as follows: rebuilding the level of 5-HT; boosting MAO activity; restoring the striatal content of DA and 3,4-Dihydroxyphenylacetic acid (DOPAC); mediating opioid and GABAergic pathways; suppressing synaptic synchronization, Ca2 overload, and presynaptic glutamate release; enhancing in the decreased level of PSD-95, Syn-l, a-Syn, p-CaMK II, CaM, and NR2B expression; augmenting the ROS production and pro-apoptosis pathways; promoting cholesterol efflux (ChE) response in macrophages by upregulating ABCA1 (ATP-binding cassette transporter A1); inhibitory influence on calpain activity; increasing the activity of LCAT; upregulating expression of LDLR protein; preserving the synaptic proteins including syn-I and a-syn and synapses function; increasing the expression of proteins involved in regulating Ca2+ influx and glutamate release such as PSD-95, p-CaMK II, CaM, and NR2B; inhibitory action against both butyrylcholinesterase (BChE) and acetylcholinesterase (AChE); reducing the increased levels of inflammatory mediators being C-reactive protein (CRP); total nuclear factor kappa-B (NF-κB); and monocyte chemoattractant protein-1 (MCP-1); reducing mRNA expression of MAPK-1 gene in hippocampus; enhancing BDNF; and depletion of pro-inflammatory mediators such as TNF-α, IL-1β, NF-κB signaling pathway, and iNOS expression.

Several neuroprotective effects, including anti-mutagenic, antioxidant, antitumor, and immunomodulatory properties, have been identified for the curry leaf tree, which are supported by the following proposed mechanisms: AChE inhibitory activity; glycation inhibitory effect; significant effect on 1,1-diphenyl-2-picrylhydrazyl (DPPH), ferric reduction, and ferrous chelating properties; scavenging activity of hydroxyl, peroxide radicals, and superoxide radicals; inhibiting the superoxide generation; inhibiting the expression of early antigen of Epstein Barr virus (EA-EBV); promoting leukocyte migration; inhibitory effects on 26S proteasome in MDA-MB231 cells; attenuating the levels of nitric oxide, inflammation-related cytokines, and other gene products, including iNOS, iCAM, NF-kB, and c-MYC; increasing phagocytic activity of macrophages.

Neuroprotective effects of fenugreek are shown in three age-related neurological diseases including dementia and Alzheimer’s disease and Parkinson’s disease. The proposed mechanisms are as follows: AChE inhibitory activity (associated with FSE-mediated activation of Akt/GSK3β pathway); enhancing the activity of antioxidant enzymes (GSH, CAT, and SOD); and reducing the levels of inflammatory mediators such as Iba-1, IL-1β, IL-6, TNF-α, iNOS, NF-κB, COX-2, CDK5-mediating tau pathology; antihyperlipidemic actions; and anti-oxidative and ROS scavenging activities.

Fennel has neuroprotective effects against Alzheimer’s disease, dementia, and multiple sclerosis, and the proposed mechanisms are as follows: acetylcholinesterase (AChE) and butyrylcholinesterase (BChE) inhibitory activity, increasing the depleted levels of oxidative stress markers (SOD1 and peroxiredoxin-6), and the three isoforms of APP (APP common, 770 and 695) expression; estrogenic function; suppression of nuclear transcription factor κB (NF-κB) pathway (by the inhibiting the inhibitory subunit of NF-kB (IκBα) degradation.

Coriander is involved in the treatment of Alzheimer’s disease and epilepsy. The proposed mechanisms are as follows: counteracting the increased intracellular production of reactive oxygen species and the activation of pro-apoptotic caspase-3 enzyme, inhibiting the receptor of epidermal growth factor and the extracellular signal-regulated kinase (ERK) phosphorylation, inhibiting lipid peroxidation, reducing LDH activity, a potential activator of neuronal voltage-gated potassium channel subfamily Q (KCNQ).

Cumin has neuroprotective effects against Alzheimer’s disease, dementia, Parkinson’s disease, and epilepsy. The mechanisms that were proposed are as follows: acetylcholinesterase (AChE) inhibitory activity, inhibiting lipid peroxidation, inhibitory effects on the fibrillation of α-SN, elevating the decreased levels of antioxidant enzyme (superoxide dismutase (SOD) and catalase), and anticonvulsant and sedation activity.

Cardamom is involved in treating Alzheimer’s disease with the following mechanisms: anti-cholinesterase activity, induction of endogenous antioxidants such as glutathione and superoxide dismutase, impeding the production of reactive hydroxyl radicals, preventing the formation of Aβ42 deposits, and protecting cells from iron-induced death.

The set of mentioned cases shows the effectiveness of the mentioned spices in the treatment of various age-related neurological diseases, and the mechanisms presented for each of them can be the subject of further studies.

The limitation of our study is the small number of randomize clinical trials. Therefore, we had to include *in vitro* and *in vivo* studies in our article, so we recommend that more clinical trial studies be done to evaluate the effect of these plants on humans. For the description of future research needs and priorities, we suggest that more *in vitro* studies be conducted to discover more mechanisms of these Indian plants on neurological disorders.
